# SLC6A8-mediated intracellular creatine accumulation enhances hypoxic breast cancer cell survival via ameliorating oxidative stress

**DOI:** 10.1186/s13046-021-01933-7

**Published:** 2021-05-14

**Authors:** Qiao Li, Manran Liu, Yan Sun, Ting Jin, Pengpeng Zhu, Xueying Wan, Yixuan Hou, Gang Tu

**Affiliations:** 1 Key Laboratory of Laboratory Medical Diagnostics, Chinese Ministry of Education, Chongqing Medical University, Chongqing, 400016 China; 2 Department of Cell Biology and Medical Genetics, Basic Medical School, Chongqing Medical University, Chongqing, 400016 China; 3 Experimental Teaching Center of Basic Medicine Science, Chongqing Medical University, Chongqing, 400016 China; 4 Department of Endocrine and Breast Surgery, the First Affiliated Hospital of Chongqing Medical University, Chongqing Medical University, #1 You-Yi Rd., Yu-zhong District, Chongqing, 400016 China

**Keywords:** SLC6A8, Creatine, Triple-negative breast cancer, Survival, Hypoxia

## Abstract

**Background:**

Triple-negative breast cancer (TNBC) is the most aggressive subtype of breast cancer, with poor prognosis and limited treatment options. Hypoxia is a key hallmark of TNBC. Metabolic adaptation promotes progression of TNBC cells that are located within the hypoxic tumor regions. However, it is not well understood regarding the precise molecular mechanisms underlying the regulation of metabolic adaptions by hypoxia.

**Methods:**

RNA sequencing was performed to analyze the gene expression profiles in MDA-MB-231 cell line (20% O_2_ and 1% O_2_). Expressions of *Slc6a8*, which encodes the creatine transporter protein, were detected in breast cancer cells and tissues by quantitative real-time PCR. Immunohistochemistry was performed to detect SLC6A8 protein abundances in tumor tissues. Clinicopathologic correlation and overall survival were evaluated by chi-square test and Kaplan-Meier analysis, respectively. Cell viability assay and flow cytometry analysis with Annexin V/PI double staining were performed to investigate the impact of SLC6A8-mediated uptake of creatine on viability of hypoxic TNBC cells. TNBC orthotopic mouse model was used to evaluate the effects of creatine in vivo.

**Results:**

SLC6A8 was aberrantly upregulated in TNBC cells in hypoxia. SLC6A8 was drastically overexpressed in TNBC tissues and its level was tightly associated with advanced TNM stage, higher histological grade and worse overall survival of TNBC patients. We found that SLC6A8 was transcriptionally upregulated by p65/NF-κB and mediated accumulation of intracellular creatine in hypoxia. SLC6A8-mediated accumulation of creatine promoted survival and suppressed apoptosis via maintaining redox homeostasis in hypoxic TNBC cells. Furthermore, creatine was required to facilitate tumor growth in xenograft mouse models. Mechanistically, intracellular creatine bolstered cell antioxidant defense by reducing mitochondrial activity and oxygen consumption rates to reduce accumulation of intracellular reactive oxygen species, ultimately activating AKT-ERK signaling, the activation of which protected the viability of hypoxic TNBC cells via mediating the upregulation of Ki-67 and Bcl-2, and the downregulation of Bax and cleaved Caspase-3.

**Conclusions:**

Our study indicates that SLC6A8-mediated creatine accumulation plays an important role in promoting TNBC progression, and may provide a potential therapeutic strategy option for treatment of SLC6A8 high expressed TNBC.

**Supplementary Information:**

The online version contains supplementary material available at 10.1186/s13046-021-01933-7.

## Background

Breast cancer (BC) is one of the most prevalent malignant tumors among women and the second major cause of female cancer-related mortality worldwide [1]. Breast cancers devoid of expression of estrogen receptor alpha (ERα), progesterone receptor (PR) and amplification of human epidermal growth factor receptor 2 (HER2) are classified as triple-negative breast cancer (TNBC), which constitutes about 15% of all breast cancers and is the most aggressive subtype of breast cancer [2]. Highly aggressive, rapidly growing solid tumors often contain hypoxic regions which are caused by the imbalance between the rapid and uncontrolled proliferation of tumor cells and inadequate blood supply. Ample evidence suggests that altered metabolic profile is induced by hypoxia to gain an undeniable survival advantage for cancer cells, ultimately limiting patient prognosis [3]. Metabolic flexibility exhibited by cancer cells under hypoxic conditions renders cancer cells addicted to certain nutrients in a way that non-transformed cells are not [4]. Identification of the molecular mechanisms mediating metabolic alteration may be useful in developing novel TNBC therapy regimen.

Cancer cells experiencing oxygen deprivation are characterized by elevated reactive oxygen species (ROS) production [5]. ROS is mainly produced as a byproduct of oxidative phosphorylation at NADH–ubiquinone oxidoreductase (complex I, CI) and ubiquinol–cytochrome c reductase (complex III, CIII) of the electron transport chain (ETC) in the mitochondria membrane [6] . The complex I is a major ROS-producing site in the mitochondria when oxygen availability declines [7]. ROS serves dual roles in cancer cells. Whereas low level of ROS plays a vital role in cancer progression, including tumor initiation, angiogenesis and metastases, excessive ROS is toxic to tumor cells and has a suppressive impact on cancer progression, leading to oxidative stress induced-cancer cell death [8]. As a result, cancer cells strive to establish a redox balance via developing numerous cellular antioxidant systems, which are chiefly achieved by metabolism reprogramming. Importantly, mitochondria are indispensable for regulation of redox balance in hypoxia [9]. It has been reported that hypoxia induces repression of complex I and III activity to reduce ROS production via miR-210/ISCU1/2 axis, leading to inhibition of apoptosis during hypoxic stress [10], suggesting that the adaptive modulation of mitochondrial functions is essential in hypoxic conditions. However, the involvement of mitochondria in tumor malignant progression and the mechanisms underlying the finetuning of mitochondrial functions to establish redox equilibrium in hypoxia remain to be elucidated.

The upregulation of nutrient transporter employed by cancer cells may be involved in maintaining redox homeostasis. For instance, SLC7A11 and SLC25A11 are induced by hypoxia to increase import of cystine and glutathione to satisfy the relatively high demand for antioxidant defense, preventing excessive ROS formation and induction of cell death in multiple human cancer cells under hypoxic conditions [11, 12]. Consistently, our RNA-seq data revealed a series of upregulated solute carrier family genes in response to hypoxia, among which *Slc6a8* was the top regulated gene. *Slc6a8* encodes a cellular membrane surface transporter (SLC6A8) controlling the uptake of creatine (Cr) into cells in a Na^+^/Cl^−^ dependent manner [13]. The enhanced SLC6A8, which serves to accumulate high amounts of intracellular Cr against a steep concentration gradient, has been found to be required for maintaining normal physiological activities of multiple high-energy consuming organs such as brain and skeletal muscle [14]. Mutation of *Slc6a8* gene results in creatine transporter deficiency (CTD), which is characterized by intellectual disability, aphasia and epilepsy [15]. Depletion of SLC6A8 reduces adipocyte creatine abundances and impairs adipocyte thermogenesis, causing obesity in mice, indicating the pivotal role of creatine in energy metabolism [16]. In tumors, previous study has demonstrated that SLC6A8 is overexpressed in human liver metastases compared with primary colorectal tumors and associated with enhanced colorectal cancer cell survival during hypoxic stress, suggesting the protective effects of SLC6A8 in cancer cells. It was recently reported that SLC6A8 knockdown suppressed the invasion and migration of human hepatocellular carcinoma cells [17]. Nonetheless, the implications of SLC6A8 in tumor progression remain poorly studied.

Creatine was initially found to have vital roles in skeletal muscle, brain and photoreceptors [18]. Lately, a resurgence of interest in creatine biology is mounting owing to the realization that this metabolite has key roles in cells beyond muscle and brain. Some cancer cells leverage creatine to support energy metabolism to fuel their survival. For instance, phosphocreatine is exploited by colorectal cancer cells to support survival upon colonization in the liver microenvironment [19]. Disruption of creatine metabolism by creatine kinase B depletion decreases cell viability and induces ovarian cancer cell apoptosis under hypoxia [20]. However, there is more to the biological roles of creatine than satisfying cellular energy needs. For example, creatine can blunt M (IFN-γ) polarization by suppressing IFN-γ-dependent proximal signaling in an energy-independent manner in macrophages [21]. Additionally, creatine is also endowed with antioxidant capacities, suggesting that creatine can serve as antioxidant to hypoxic cancer cells. However, fairly little is known regarding how tumor cells utilize creatine to combat oxidative stress and acquire a survival advantage under hypoxia.

Herein, we show that SLC6A8 is robustly induced by p65/NF-κB in hypoxic TNBC cells and aberrantly overexpressed in TNBC tumor tissues. The enhanced SLC6A8 is closely associated with advanced TNM stage, histological grades and poor prognosis of TNBC patients. Functionally, we unravel that SLC6A8 contributes to increased intracellular creatine pool, which promotes hypoxic TNBC cell survival and inhibits cell apoptosis by bolstering antioxidant capability and activating AKT-ERK signaling.

## Materials and methods

### Clinical samples

The samples of tumor tissues and their normal counterparts analyzed in this study were obtained from breast cancer patients with no previous history of radiotherapy or chemotherapy at the First Affiliated Hospital of Chongqing Medical University. The investigation was approved by the ethics committee of Chongqing Medical University beforehand.

### Cell culture

Human breast cancer cells (MDA-MB-231, BT549, SKBR3, Hs578T, BT-474, MDA-MB-468, SUM-159, MDA-MB-453 and MCF-7) were obtained from American Type Culture Collection (ATCC). Those cells were routinely cultured in DMEM or RPMI 1640 (Gibco-BRL, Australia) comprising 10% fetal bovine serum (FBS; Gibco BRL, Australia), 100 U/ml penicillin and 100 mg/ml streptomycin. For hypoxic cell culture, cells were cultured at 37 °C in a humidified tri-gas incubator containing 5% CO_2_ and 1% O_2_. Controls were cultured at 37 °C in a standard humidified incubator containing 5% CO_2_ and 20% O_2_. For creatine pretreatment, cells were cultured in media containing 1% FBS and 5 mM creatine (Sigma-Aldrich, USA) 24 h ahead of experiments.

### Cell transfection, plasmids and reagents

Short hairpin RNA (shRNA) oligonucleotides targeting *Slc6a8*, *p65/NF-κB*, *HIF1A* and *HIF2A*, and control shRNA-NC were all purchased from GenePharma (Shanghai, China). The breast cancer cells (MDA-MB-231 and BT-549) that stably expressed above shRNAs were established by lentivirus transduction according to the manufacturer’s instructions. The sequences of shRNA used were listed in Supplementary Table 1. The promoter containing TP53/FOS/ETV4/p65/NF-κB-wild type binding sites (WT) or mutated binding sites (MUT) was cloned into pGL3 luciferase reporter vector to obtain the pGL3/*Slc6a8* WT reporter and pGL3/*Slc6a8* MUT reporter (GenePharma, China). The AKT inhibitor Capivasertib (AZD5363) was purchased from Selleckchem (USA).

### RNA isolation and quantitative real-time PCR

Total RNAs of cells or tissue samples were extracted with TRIzol (Invitrogen, USA). The cDNA was synthesized from the purified RNA by using PrimeScript RT Reagent Kit (TaKaRa, Japan). The qRT-PCR was conducted on a Bio-Rad CFX96 system using the SYBR Premix Ex Taq™ system (TaKaRa) following the manufacturer’s protocols. The relative gene expression was calculated using the 2^-ΔΔCT^ method and normalized to respective β-actin levels. The primer sequences used in qRT-PCR were listed in Supplementary Table 2.

### RNA sequencing

The total RNA was extracted from MDA-MB-231 cells that were cultured under hypoxia or normoxia for 24 h using TRIzol reagent (Invitrogen). Subsequently, RNA was purified through rRNA depletion and then subjected to cDNA synthesis and RNA amplification. Next, random hexamer primer cDNA libraries were sequenced on Illumina Hiseq 4000 sequencing platform following the manufacture’s instructions for paired-end 150 bp reads (Lifegenes, Shanghai, China). HTSeq v0.6.1 was used to count the reads numbers mapped to each gene. Differential expression analysis of two samples was performed using the DEGseq (2010) R package. The raw RNA-seq data were submitted to the Sequence Read Archive database (SRA) (accession number: PRJNA708216).

### Dual-luciferase reporter assay

pGL3/*Slc6a8* wild-type (WT) reporter, pGL3/*Slc6a8* MUT reporter with mutated FOS, TP53, ETV4 or p65/NF-κB binding sites in the *Slc6a8* promoter or control pGL3 reporter was transfected into cells using Lipofectamine 2000 (Invitrogen), and pRL-TK reporter vector was used as control. The cells were cultured under normoxia or hypoxia for 24 h. Cell lysates were collected and a Dual-Luciferase Reporter Assay System (Promega, USA) was used to analyze the luciferase activity.

### Chromatin immunoprecipitation (CHIP)

ChIP assays were conducted using a ChIP kit (Thermo, USA) following the manufacturer’s instructions. Briefly, cells were crosslinked with 1% formaldehyde (15 min), quenched in 125 mM glycine (5 min) and sonicated to shear chromatins into DNA fragments. Next, immunoprecipitation was performed by adding p65/NF-κB and control IgG antibodies to pull down target proteins. The target protein was digested with Proteinase K and then the precipitated DNA was purified using QIAquick PCR Purification Kit (Qiagen, German) and subsequently quantified by qRT-PCR. The primer sequences used in qRT-PCR to amplify the fragment of p65/NF-κB-binding motif in the *Slc6a8* promoter region were listed in Supplementary Table 2.

### Creatine quantification assay

Creatine concentration was measured by using Creatine Assay Kit (Abcam, UK) following the manufacturer’s instructions. For cell samples, cells (2 × 10^6^) were collected and washed with cold PBS. For tumor tissue samples, appropriate amount of tissue was harvested and washed with PBS. Then, the supernatants were deproteinized, mixed with reaction mixture and incubated at 37 °C for 1 h. The optical density (OD 570 nm) representing creatine concentrations was detected by EON spectrophotometer (BioTek, USA). The total amount of creatine was normalized to cell numbers.

### Western blot analysis

Total proteins were extracted using RIPA lysis buffer (Beyotime, China) and quantified using the BCA protein assay kit (Beyotime, China). Proteins of different molecular weight was separated using 8–12% SDS-PAGE gels via electrophoresis. Then proteins were transferred from the gels to 0.22 μM PVDF membranes (Bio-Rad, CA, USA). 5% skim milk powder was used to block the PVDF membranes at room temperatures for 2 h and incubated with specific primary antibodies against SLC6A8 (Abcam, Cat. No.: 62196, 1:1000), p65/NF-κB (CST, Cat. No.: 8242, 1:1000), HIF1A (CST, Cat. No.: 14179, 1:1000), HIF2A (CST, Cat. No.: 7096, 1:1000), p-AKT (CST, Cat. No.: 4060, 1:1000), AKT (CST, Cat. No.: 4691, 1:1000), p-ERK1/2 (CST, Cat. No.: 9101, 1:1000), ERK1/2 (CST, Cat. No.: 4695, 1:1000), Bcl-2 (CST, Cat. No.: 15071, 1:1000), Bax (CST, Cat. No.: 5023, 1:1000), cleaved Caspase-3 (CST, Cat. No.: 9661, 1:1000), Ki-67 (SAB, Cat. No.: 48871, 1:1000) and β-Actin (CST, Cat. No.: 4970, 1:1000) at 4 °C overnight. Next, the membranes were incubated with appropriate secondary antibodies at room temperatures for 2 h. The protein bands were visualized using the enhanced chemiluminescence system (Amersham Pharmacia Biotech, Japan). β-Actin was used as control. Blots were stripped by stripping buffer (CWBIO, China) when needed.

### Immunohistochemistry (IHC) analysis

Paraffin-embedded tissue sections were used to perform IHC staining according to the manufacturer’s protocols. Briefly, following dewaxing and hydration, the sections were incubated with 3% hydrogen peroxide to block endogenous peroxidase and then heated in a microwave for antigen retrieval (AR), after which they were incubated with primary antibodies against SLC6A8 (1:100), Bcl-2 (1:100), Bax (1:100), cleaved Caspase-3 (1:100), Ki-67 (1:100) at 4 °C overnight, followed by secondary antibody incubation at room temperature for 1 h. Then, the sections were stained with diaminobenzidine (DAB) and counterstained with hematoxylin. The images were captured using eclipse 80i (Nikon, Japan).

### Cell viability assay

Cell viability assay was performed using Cell Counting Kit-8 (Solarbio, China). 3000 cells/well were seeded in wells of 96-well plates at a volume of 100 μl/well and treated as indicated. 10 μl CCK8 solution was added into each well and incubated in the dark at 37 °C for 2 h. The optical density (OD 450 nm) representing cell viability was measured with EON spectrophotometer (BioTek, USA).

### Cell apoptosis assay

Cells were collected by EDTA-free trypsin and resuspended in 500 μl cold PBS at a density of 2 × 10^6^ cells/ml after centrifugation. Cells were double-stained with fluorescein isothiocyanate (FITC)-conjugated Annexin V and propodium iodide (PI) and then examined on a flow cytometer (BD FACSCalibur, USA).

### Measurement of the mitochondrial membrane potential (MMP, *ΔΨm*)

The mitochondrial membrane potential was measured using mitochondrial membrane potential assay kit with JC-1 (Beyotime, China) following the manufacturer’s protocols. Briefly, cells were seeded into 6-well plates. Cells (1 × 10^6^) were washed with PBS and incubated in JC-1 working solution in the dark at 37 °C for 20 min. The images were captured by using eclipse Ti (Nikon, Japan) and the intensity of red fluorescence (Ex/Em = 525/590 nm) representing JC-1 aggregates was determined by using Cary Eclipse (Agilent).

### Analysis of mitochondrial respiratory chain complex activity

The activities of the mitochondrial complex I or III were assessed by using the commercially available kits (Solarbio, China) following the manufacturer’s instructions. Briefly, mitochondrial homogenates were isolated from a total of 5 × 10^6^ cells and instantly mixed with the corresponding reaction buffer. The enzymatic activities of the mitochondrial complex I and complex III were then determined by measuring absorbance of reaction mixture at 340 nm or 550 nm using UV-2550 (Shimadzu, Japan), respectively.

### Oxygen consumption rate (OCR) measurement

The mitochondrial OXPHOS was monitored by detecting the OCR using a Seahorse XF24 Extracellular Flux Analyzer (Seahorse Bioscience, USA). Briefly, 2 × 10^4^ cells were seeded into XF24 cell culture microplates. Before the detection, cells were changed into XF Base Media. Then, oligomycin (2 μM), carbonyl cyanide 4-(trifluoromethoxy) phenylhydrazone (FCCP) (1.5 μM), rotenone (Rot) (2 μM) and antimycin A (AA) (2 μM) were successively added for the determination of OCR parameters.

### Detection of intracellular reactive oxygen species (ROS)

The intracellular ROS level of TNBC cells and tissues was detected using ROS Assay Kit (Beyotime) according to the manufacturer’s protocols. Briefly, cells were washed with PBS and incubated in serum-free media containing 10 μM chloromethyl-2′,7′-dichlorodihydrofluorescein diacetate (DCFH-DA) in the dark for 20 min. Then, trypsinized cells were washed with PBS twice and fluorescent images were captured by using eclipse Ti (Nikon, Japan). The fluorescent intensity (Ex/Em = 488/525 nm) was determined by using Cary Eclipse (Agilent). For in vivo ROS measurement, tumor tissues were harvested from mice and dissociated with GentleMACS (Miltenyi, German), and then single cell suspensions were stained with 10 μM DCFH-DA for determination of fluorescent intensity.

### Tumor xenograft models

The animal experiments were conducted with the approval of animal care ethics committee of Chongqing Medical University. The MDA-MB-231/*shSlc6a8* or MDA-MB-231/shNC cells were subcutaneously inoculated into 4-week female nude mice (1 × 10^5^ cells per mouse) randomly. For mouse with exogenous creatine supplementation, 100 ml sterilized creatine solution (0.25 M)/mouse was intraperitoneally injected into the mice daily for a week prior to cell injection and the whole time until the termination of the animal study. The mice supplemented with sterile PBS worked as controls. Tumor volumes were measured with a vernier caliper at an interval of 7 days starting a week after injection and calculated as 0.5 × length×(width)^2^. 4 weeks after the inoculation, the mice were sacrificed and the xenografted tumors were excised and weighed. And the tumor masses were subjected to further analysis.

### TCGA database analysis

In our analysis, the RNA-seq data of 965 breast cancer specimens, 112 adjacent normal tissue specimens and corresponding clinical data were downloaded from The Cancer Genome Atlas (TCGA, https://cancergenome.nih.gov/). The expression of genes was uniformly determined as Transcripts Per Kilobase of exon model per Million mapped reads (TPM).

### Statistical analysis

Statistical analysis was performed using SPSS 20.0 software and GraphPad Prism 6.0. The differences among groups were assessed using Student’s t-test (one-factor two groups), one-way ANOVA test (one-factor three or more groups) or chi-square test. Calculation of a receiver operating characteristic curve (ROC) and the Kaplan–Meier analysis were performed to assess the diagnostic value and patients’ overall survival (OS) rate, respectively. Pearson’s correlation coefficient was applied to test for correlation between the expression of *Slc6a8* and other RNAs. All experiments were independently repeated at least three times and data were presented as mean ± standard deviation (SD). A value of *P* < 0.05 was considered as statistically significant.

## Results

### SLC6A8 is upregulated in response to hypoxia in TNBC cells

**Fig. 1:**
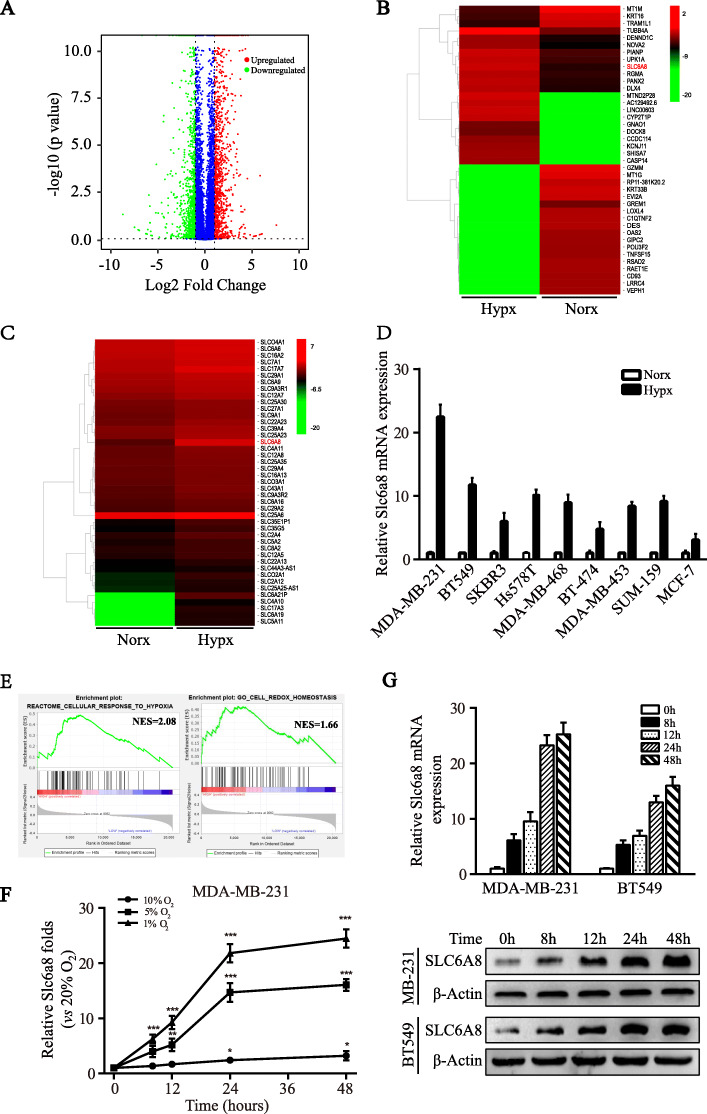
RNA expression profiles of hypoxic TNBC cells. **a** The volcano plot visualized RNA expression of TNBC cell MDA-MB-231 exposed to hypoxia (Hypx, 1% O_2_) vs normoxia (Norx, 20% O_2_) for 24 h. The red and green dots represented the significantly upregulated and downregulated RNAs, respectively. **b** A heat map showed the 50 most upregulated and downregulated RNAs in hypoxic MDA-MB-231 cells. **c** A heat map showed the upregulated RNAs encoding solute carrier family transporters. **d** The hypoxic induction of *Slc6a8* mRNA expression in various breast cancer cells was detected by qRT-PCR. **e** Gene set enrichment analysis (GSEA) showed that *Slc6a8* expression was positively associated with cellular response to hypoxia and redox homeostasis. **f** Time- and oxygen concentration-dependent upregulation of *Slc6a8* expression in MDA-MB-231 was detected by qRT-PCR. **g** Time dependent upregulation of *Slc6a8* mRNA and SLC6A8 protein expressions in MDA-MB-231 and BT549 cells were validated by qRT-PCR and western blot, respectively. Data were presented as mean ± SD (^*^P < 0.05, ^**^P < 0.01, ^***^P < 0.001)

As a common existence in solid tumors, hypoxia is tightly associated with tumor progression and often leads to enhanced local invasiveness, altered metabolism, unregulated angiogenesis, incipient metastases and spread of cancer stem cells [22]. In an effort to better understand the adaptations elicited by hypoxia in TNBC, we performed RNA-seq to compare the distinct gene profiles of TNBC cell MDA-MB-231 under normoxia (Norx, 20% O_2_) and hypoxia (Hypx, 1% O_2_) condition. With a cut-off value set as “fold change > 2.0 and *P* < 0.05”, we found 2255 differentially regulated genes, including 1210 upregulated genes and 1045 downregulated genes, as presented in the volcano plot (Fig. 1a). The top 50 increased and decreased genes were depicted by heatmap (Fig. 1b). Remarkably, we found that many of these hypoxia-induced genes belong to the solute carrier family (Fig. 1c). To validate the results of RNA-seq analysis, we randomly selected 15 obviously upregulated RNAs and 15 significantly upregulated RNAs encoding solute carrier transporters (*P* < 0.01) to detect their expressions in normoxic and hypoxic MDA-MB-231 cells by qRT-PCR, and *Slc6a8* was found to be most upregulated by hypoxia (Figure S1A-B). Then, the upregulation of *Slc6a8* was further confirmed in a set of different breast cancer cell lines that were cultured under normoxia or hypoxia for 24 h (Fig. 1d). Importantly, *Slc6a8* was substantially upregulated in hypoxic TNBC cells rather than non-TNBC cells, indicating the preferential upregulation of *Slc6a8* expression in hypoxic TNBC cells. Moreover, gene set enrichment analysis (GSEA) was performed to detect the potential biological functions of SLC6A8, and it was found that *Slc6a8* was positively related to cell biological processes of cellular response to hypoxia and cell redox homeostasis (Fig. 1e). Next, we investigated the hypoxic response of *Slc6a8* gene. As oxygen concentration gradually decreased, we found that the *Slc6a8* mRNA expression level was increased in a time-dependent manner (Fig. 1f). And 1% oxygen concentration also aberrantly induced upregulation of RNA and protein expression of SLC6A8 as the treatment time increased in MDA-MB-231 and BT549 cells (Fig. 1g). These data collectively suggest that hypoxia induces SLC6A8 upregulation in TNBC cells.

### The enhanced SLC6A8 is closely associated with TNBC progression

**Fig. 2:**
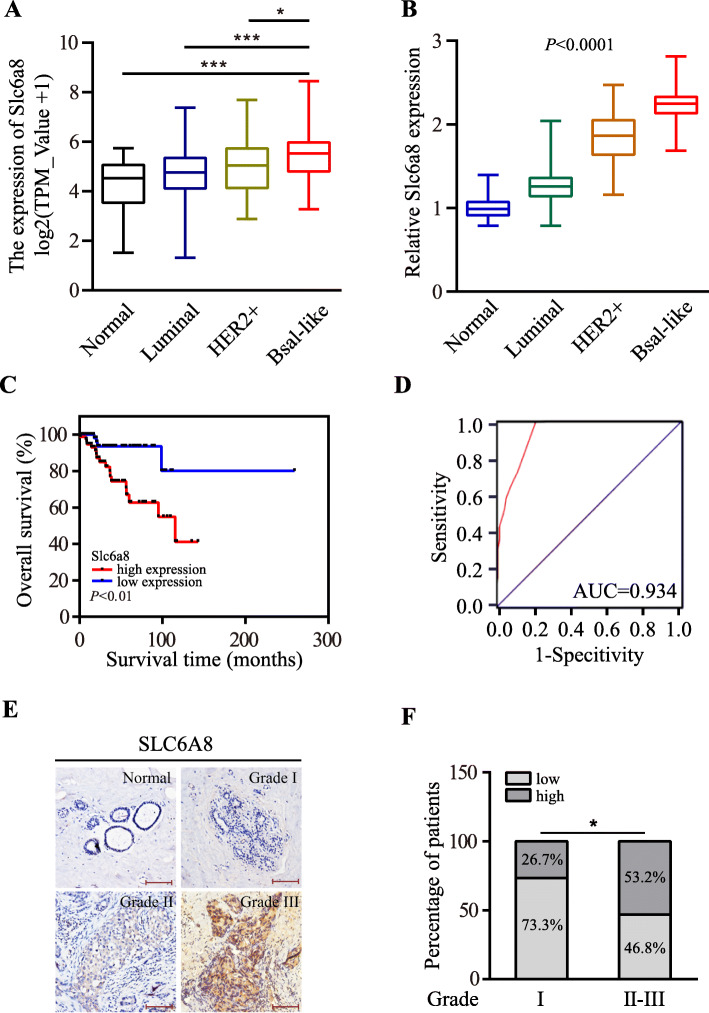
SLC6A8 expression is upregulated in TNBC tissues and associated with TNBC clinical progression. **a** Analysis of *Slc6a8* expression in human breast cancers by tumor subtype based on data obtained from TCGA database (Normal, *n* = 112; Luminal, *n* = 815; HER2, *n* = 36; Basal-like TNBC, *n* = 114). **b** *Slc6a8* mRNA expression was detected by qRT-PCR in human breast cancer tissues and normal counterparts (Normal, *n* = 60; Luminal, *n* = 67; HER2, *n* = 22; Basal-like TNBC, *n* = 60). **c** Kaplan-Meier survival analysis of the relationship between *Slc6a8* expression and overall survival of TNBC patients based on TCGA data. **d** The ROC curve indicated the diagnostic value of SLC6A8 for TNBC. **e** Representative images of SLC6A8 abundances in adjacent non-cancerous tissues and TNBC tissues of different histopathological grades (Scale bars, 100 μm). **f** Analysis of the ratio of low or high SLC6A8 protein levels according to histological grades of 60 TNBC samples. (^*^P < 0.05, ^***^P < 0.001)

Next, we wondered what the potential relationship was between SLC6A8 expression and clinical TNBC progression. By analysis of transcriptome data of breast cancer tissues and para-cancerous tissues obtained from TCGA database, we found that the expression of *Slc6a8* gene was substantially upregulated in breast cancer tissues as compared to adjacent normal tissues (Fig. 2a), which was further confirmed by qRT-PCR in a cohort of 149 breast cancer patients (Fig. 2b). Of note, as shown in Fig. 2a and b, the highest level of SLC6A8 existed in malignant basal-like breast cancer (TNBC) in comparison with other breast cancer subtypes. Interestingly, based on analysis of the microarray data derived from Oncomine database (https://www.oncomine.org/resource/login.html), we unraveled an enhanced SLC6A8 expression in other solid cancers, such as bladder cancer, colorectal cancer, esophageal squamous cell carcinoma, clear cell renal cell carcinoma, squamous cell lung carcinoma and melanoma (Figure S2), suggesting a popularly enhanced SLC6A8 expression in solid tumors. Furthermore, the higher level of SLC6A8 was closely associated with worse overall survival in TNBC patients (Fig. 2c). Moreover, the ROC analysis suggested that SLC6A8 expression had great potential to sensitively distinguish TNBC patients (Fig. 2d). In addition, increased SLC6A8 expression was found to be associated with worse TNBC histological grade using IHC staining (Fig. 2e-f). Analysis of the relationship between SLC6A8 proteins and clinical characteristics of TNBC patients further confirmed that SLC6A8 level significantly related with increased TNBC incidence at younger age (*P* = 0.004), T stage (*P* = 0.01), N stage (*P* = 0.018) and TNM stage (*P* = 0.001) (Table 1) by performing chi-square test. Taken together, increased SLC6A8 expression is potentially correlated with TNBC progression.

**Table 1:** The relationship between Slc6a8 expression and clinicopathological features of TNBC patients

Characteristic		All cases	SLC6A8		Chi-square	*P* value
			Low	High		
All cases		60	30	30		
Age	<50	25	7	18	8.297	0.004^**^
	≥50	35	23	12		
T stage	T1	28	19	9	6.696	0.01^*^
	T2/3/4	32	11	21		
N stage	N0/N1	35	22	13	5.554	0.018^*^
	N2/N3	25	8	17		
TNM stage	I/II	23	17	5	10.331	0.001^**^
	III/IV	38	13	25		

^*^ *P* < 0.05, ^**^ *P* < 0.01

### Hypoxia-induced SLC6A8 expression is dependent on p65/NF-κB signaling

**Fig. 3:**
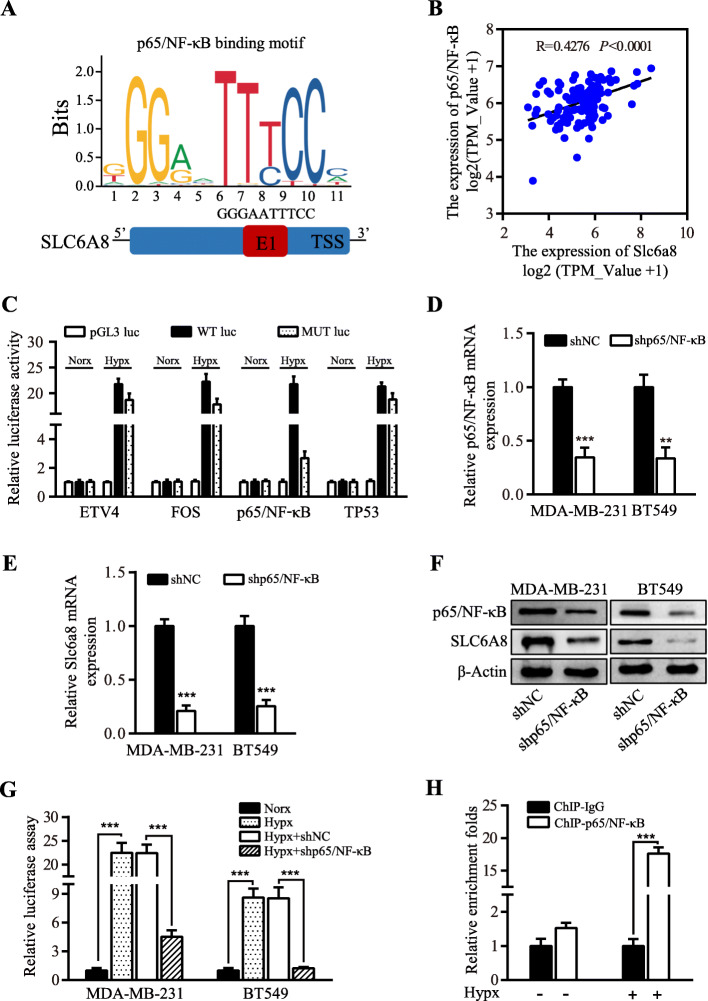
SLC6A8 is upregulated by p65/NF-κB in hypoxic TNBC cells. **a** Schematic illustration of the predicted binding sites for p65/NF-κB in the *Slc6a8* promoter region recognized from JASPAR database. **b** Pearson correlation analysis of *Slc6a8* and *p65/NF-κB* expression based on data from TCGA database. **c** MDA-MB-231 cells were transfected with pGL3-vector, pGL3/*Slc6a8* promoter wide-type (WT) reporter and pGL3/*Slc6a8* promoter mutated (MUT) ETV4, FOS, p65/NF-κB or TP53 binding sites reporter under normoxia or hypoxia for 24 h, and luciferase activity assay was performed to assess *Slc6a8* transcript activities under normoxic and hypoxic conditions. **d-f** qRT-PCR and western blot to show p65/NF-κB knockdown (**d**) decreased SLC6A8 expression both in RNA (**e**) and protein (**f**) levels in hypoxic MDA-MB-231 and BT549 cells. **g** Luciferase assay weas performed to check *Slc6a8* transcript activities in p65/NF-κB parental or silenced MDA-MB-231 and BT549 cells cultured in normoxia or hypoxia condition for 24 h. **h** ChIP assay was conducted to measure the binding of p65/NF-κB to the *Slc6a8* promoter region in MDA-MB-231cells cultured in normoxia and hypoxia by p65/NF-κB and IgG antibodies, and qRT-PCR was performed to determine the binding fragment of *Slc6a8* promoter precipitated by p65/NF-κB antibodies, presented as enrichment folds normalized to normoxia. Data were presented as mean ± SD (^**^P < 0.01, ^***^P < 0.001)

As a master regulator of hypoxic response in cells and tissues, HIF1A/2A has been believed to modulate the expression of most hypoxia-related genes [23]. To understand whether HIF1A or HIF2A was involved in regulating the hypoxic SLC6A8 expression, we generated HIF1A/2A-silenced engineered MDA-MB-231 and BT549 cells (Figure S3A-B). Interestingly, knockdown of HIF1A or HIF2A failed to affect the expressions of *Slc6a8* mRNA and SLC6A8 protein (Figure S3C-D), which was further proved by Pearson correlation analysis between *Slc6a8* and *HIF1A* or *HIF2A* expression in TNBC patients from TCGA database (Figure S3E-F). Next, to understand hypoxia-mediated upregulation of SLC6A8, we performed bioinformatics analysis to identify potential transcription factors (TFs) of *Slc6a8* gene using Promo Alggen database (http://alggen.lsi.upc.es/) and JASPAR (http://jaspar.genereg.net/). TFs that were upregulated by hypoxia and the predicted regulators of *Slc6a8* were compared, and 4 overlapping TFs (ETV4, FOS, TP53 and p65/NF-κB) were identified (Fig. 3a and Figure S4A). Coexpression analysis between *Slc6a8* with *ETV4*, *FOS*, *TP53* or *p65/NF-κB* expression from TCGA database showed that *Slc6a8* expression was exclusively in significantly positive correlation with that of *p65/NF-κB* (Fig. 3b and Figure S4B-D). And *p65/NF-κB* expression was most increased in the hypoxic MDA-MB-231 and BT549 cells among the four predicted TFs as detected by qRT-PCR (Figure S3E). Moreover, the results of the luciferase activity analysis showed that the p65/NF-κB binding sites mutation rather than ETV4, FOS or TP53 binding sites mutation in the *Slc6a8* promoter markedly abolished transcript activities of *Slc6a8* under hypoxia (Fig. 3c). Collectively, p65/ NF-κB was the optimal transcription factor of *Slc6a8*. Of note, p65/NF-κB activation was previously reported to be another critical component in the transcriptional response to hypoxia [24]. To confirm whether p65/NF-κB was involved in regulating SLC6A8 expression in hypoxic MDA-MB-231 and BT549 cells, we established p65/NF-κB stably knocked down MDA-MB-231 and BT549 cells using lentivirus-mediated p65/NF-κB directed shRNA (Fig. 3d). Importantly, the silencing of p65/NF-κB significantly reduced the expression of SLC6A8 in hypoxic TNBC cells (Fig. 3e-f). Moreover, using these engineered cells, we confirmed that hypoxia treatment substantially increased the transcript activities of *Slc6a8* checked by luciferase assay as compared to normoxia treatment, while knockdown of p65/NF-κB notably decreased the transcript activities of *Slc6a8* induced by hypoxia treatment (Fig. 3g). We then performed chromatin immunoprecipitation assay (CHIP) to further verify the binding of p65/NF-κB to the predicted sequence in the *Slc6a8* promoter region (Fig. 3h), and these results suggest that p65/NF-κB directly regulates SLC6A8 expression under hypoxia conditions.

### SLC6A8-mediated intracellular creatine accumulation promotes hypoxic TNBC cell survival

**Fig. 4:**
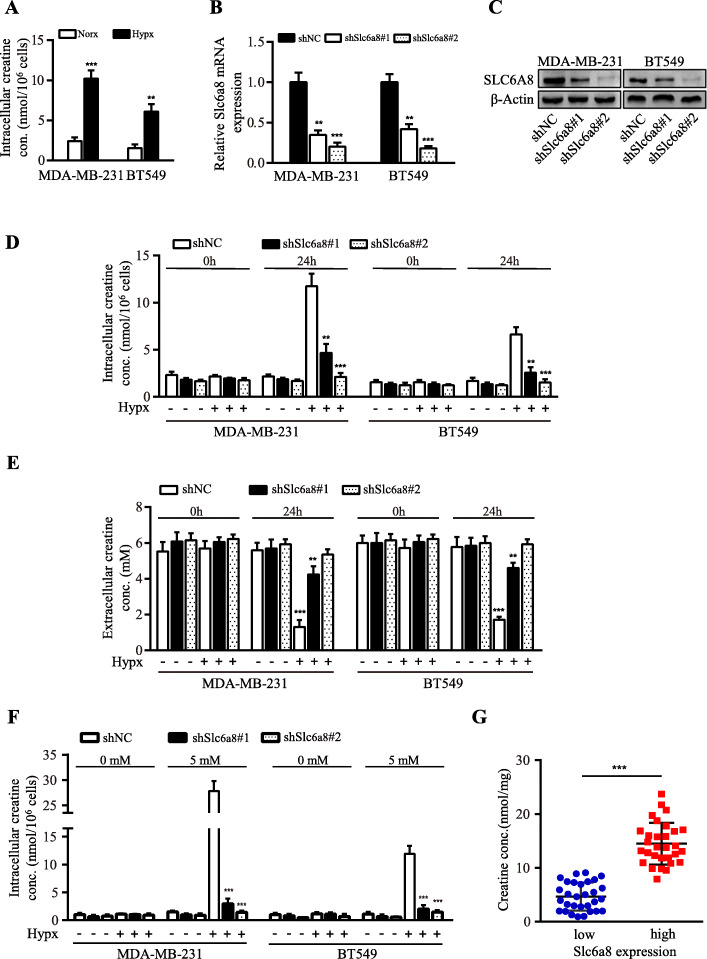
SLC6A8 mediates intracellular creatine accumulation in hypoxic TNBC cells. **a** Intracellular creatine concentrations in hypoxic and normoxic MDA-MB-231 and BT549 cells. **b, c** Lentivirus-mediated shRNA against *Slc6a8* (shSlc6a8) or scrambled control (shNC) was stably expressed in TNBC cells, the knockdown efficiencies of SLC6A8 were verified by qRT-PCR (**b**) and western blot (**c**). **d** Intracellular creatine concentrations were determined in shNC and shSlc6a8 TNBC cells cultured in media containing 10% FBS under normoxia or hypoxia for 24 h. **e** Determination of creatine concentrations in the media derived from normoxic or hypoxic MDA-MB-231 and BT549 cells transfected with shNC or shSlc6a8. **f** The *Slc6a8* parental or silenced TNBC cells that were cultured in media containing 1% FBS were additionally administered creatine (5 mM) or PBS under normoxia or hypoxia condition for 24 h, and intracellular creatine concentrations were determined. **g** Determination of intracellular creatine concentrations in tumor tissues of TNBC patients based on high and low expression of *Slc6a8* mRNA level as stratified by relative median value of *Slc6a8* mRNA level in TNBC tissues. Data were presented as mean ± SD (^**^P < 0.01, ^***^P < 0.001)

**Fig. 5:**
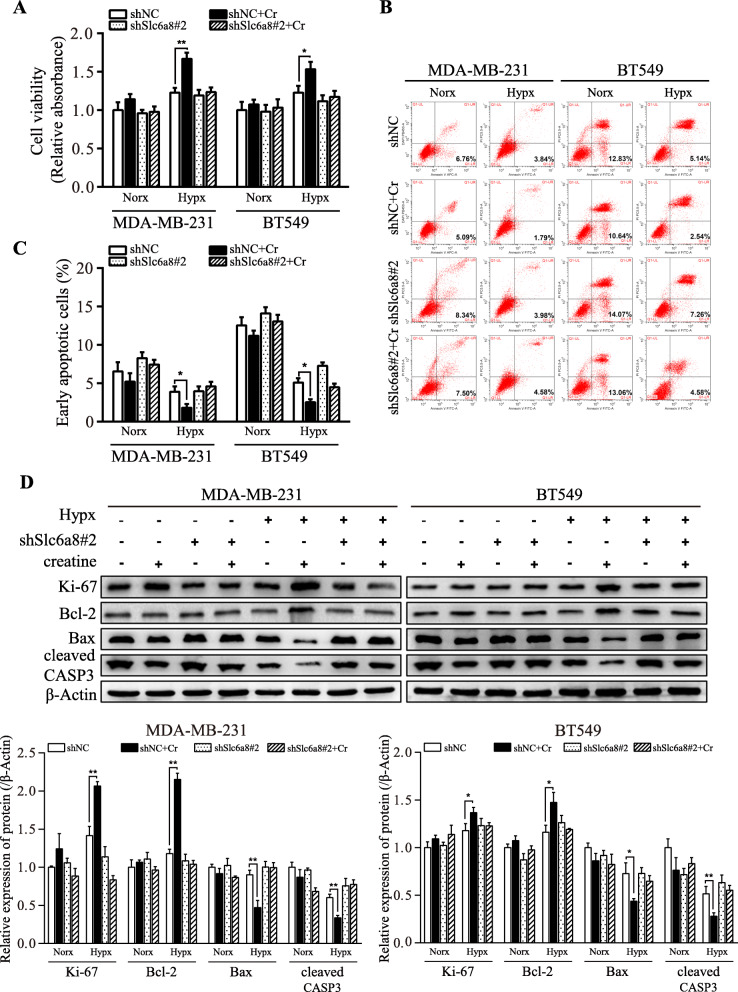
SLC6A8-mediated creatine accumulation promotes TNBC cell survival under hypoxia. shNC and shSlc6a8 TNBC cells were treated with or without 5 mM creatine (Cr) and exposed to normoxia or hypoxia for 24 h, then cell viability and apoptosis were assessed. **a** Cell viability was determined by CCK8 assay. **b, c** Cell apoptotic rate was determined using Annexin V/PI double staining. **d** The levels of Ki-67, Bcl-2, Bax and cleaved Caspase-3 (cleaved CASP3) were detected by western blot. Data were presented as mean ± SD (^*^P < 0.05, ^**^P < 0.01)

In accordance with hypoxic induction of SLC6A8, intracellular creatine levels were significantly elevated in hypoxic TNBC cells (Fig. 4a). It has been reported that creatine could promote oligodendrocyte survival under inflammatory conditions [25]. Thus, SLC6A8 loss-of-function cell models of MDA-MB-231 and BT549 were established (Fig. 4b-c). Loss of SLC6A8 led to drastic reduction of intracellular creatine concentrations in hypoxic MDA-MB-231 and BT549 cells (Fig. 4d-e). In order to eliminate the interference of creatine from FBS, cells were cultured in media containing only 1% FBS, and exogenously supplemented creatine could significantly increase intracellular creatine concentrations in *Slc6a8*-parental MDA-MB-231 and BT549 cells but not in *Slc6a8*-silenced MDA-MB-231 and BT549 cells (Fig. 4f), suggesting that SLC6A8 was crucial for maintaining high intracellular creatine amounts in hypoxic MDA-MB-231 and BT549 cells. Consistently, TNBC tissues with higher expression of SLC6A8 displayed higher level of creatine in comparison with those with lower SLC6A8 expression (Fig. 4g). Consistent with the changes of intracellular creatine concentrations, we detected increased cell viability (Fig. 5a) and lower apoptotic rates (Fig. 5b-c) of tumor cells with exogenous creatine compared to those without under hypoxia condition. Similarly, the cell viability-associated protein (e.g. Ki-67 and Bcl-2) and apoptosis-related proteins (e.g. Bax and cleaved Caspase-3) levels exhibited the same trend in these cells (Fig. 5d). Collectively, these data confirm the important role of SLC6A8-mediated creatine accumulation in supporting TNBC cell survival under hypoxia.

### Creatine mitigates excessive ROS production via reducing mitochondrial activity and oxygen consumption in hypoxic TNBC cells

**Fig. 6:**
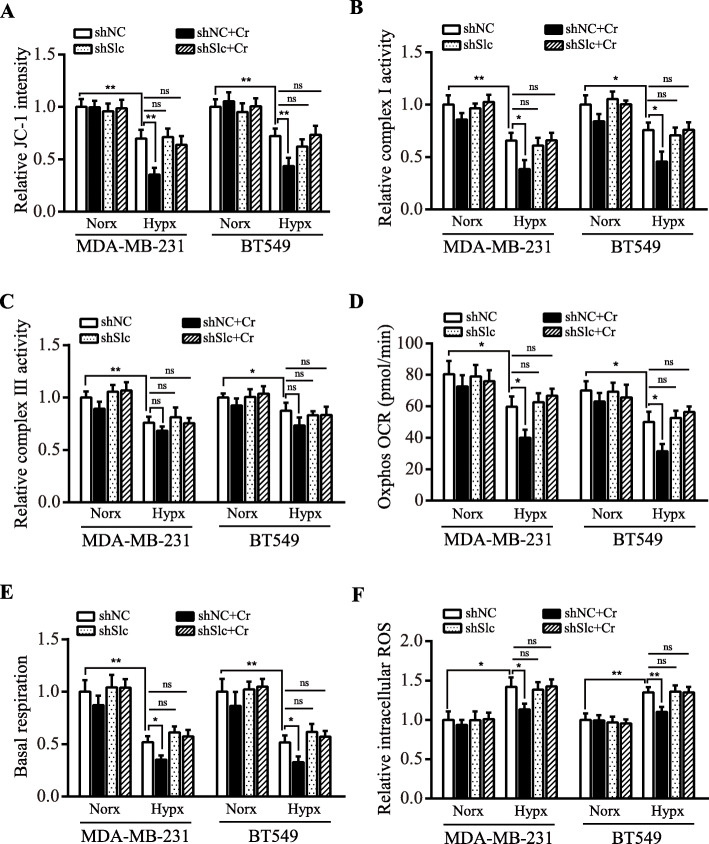
Creatine reduces mitochondrial activity and oxygen consumption, and mitigates ROS production in hypoxic TNBC cells. shNC and shSlc6a8#2 (shSlc) MDA-MB-231 and BT549 cells were cultured under normoxia and hypoxia, and administered 5 mM creatine or PBS, then mitochondrial activity, oxygen consumption and ROS products were assessed. **a** Mitochondrial membrane potential was determined by JC-1 staining. **b, c** Mitochondrial complex I and III activities were measured using reagent kit, as elaborated in methods. **d, e** Oxygen consumption rate (OCR) and basal respiration (complex I mediated OCR) were detected using a Seahorse XF24 Extracellular Flux Analyzer. **f** ROS level was measured by DCFH-DA staining. Data were presented as mean ± SD (^*^P < 0.05, ^**^P < 0.01, ^***^P < 0.001, ns means no statistical difference)

It has been known that there is a shortage of the ultimate electron acceptor O_2_ and inefficient pass of electrons through the ETC of mitochondria, incurring a burst in ROS under hypoxia [26]; and CI was reported to account for decreased oxygen consumption under hypoxia [27]. Given that creatine possesses antioxidant properties [28], we therefore speculated that creatine might play a preventive role in mitochondrial ROS accumulation by decreasing mitochondrial activity. To investigate whether creatine could suppress ROS production via reducing mitochondrial activity, JC-1 probe staining was carried out. As shown in Fig. 6a and Supplementary Figure 5A, the mitochondrial activity was reduced following exposure to hypoxia and exogenous supply of creatine could further decrease its activity in hypoxia. Next, we measured mitochondrial CI and CIII activity. Hypoxia induced a decrease in both CI and CIII activities (Fig. 6b-c). However, creatine supplementation mainly decreased CI rather than CIII activity in hypoxic TNBC cells (Fig. 6b-c). In line with above results, mitochondrial oxygen consumption rate and complex I-mediated basal respiration were significantly decreased by exogenous addition of creatine under hypoxia (Fig. 6d-e). Next, we assessed the intracellular ROS level by chloromethyl-2′,7′-dichlorodihydrofluo-rescein diacetate (DCFH-DA), the ROS indicator. ROS contents were significantly increased in hypoxic TNBC cells compared to normoxic TNBC cells, but the redox balance in hypoxic cells was restored by creatine supplementation under hypoxic condition (Fig. 6f and Figure S5B). These data show that creatine can alleviate oxidative stress under hypoxic conditions.

### Intracellular creatine enhances cell survival via activation of AKT-ERK1/2 signaling

**Fig. 7:**
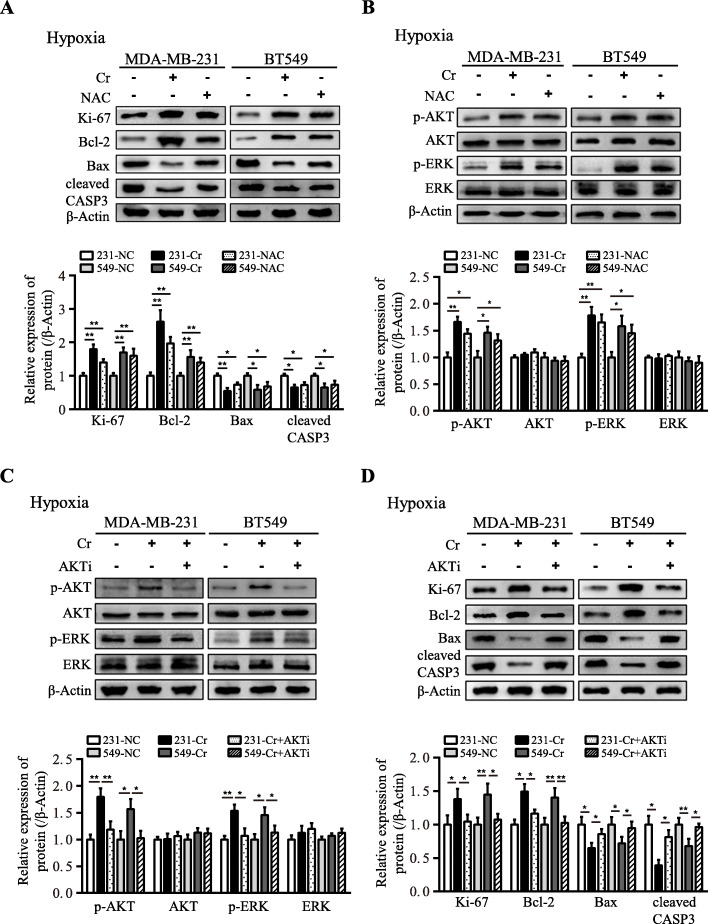
The enhanced creatine promotes hypoxic TNBC survival via attenuation of ROS-depended repression of AKT-ERK1/2 signaling. **a** Ki-67, Bcl-2, Bax and cleaved Caspase-3 (cleaved CASP3) protein levels were detected by western blot in hypoxic TNBC cells supplemented with creatine or NAC. **b** p-ERK, ERK, p-AKT and AKT protein levels were determined by western blot in hypoxic TNBC cells supplemented with creatine or NAC. **c** p-ERK, ERK, p-AKT and AKT protein levels were detected by western blot in hypoxic TNBC cells under treatment with creatine alone or combined with AKT inhibitor AZD5363. **d** Ki-67, Bcl-2, Bax and cleaved Caspase-3 (cleaved CASP3) protein levels were determined by western blot in hypoxic TNBC cells treated the same as e. Data were presented as mean ± SD (^*^P < 0.05, ^**^P < 0.01)

The aberrantly high level of ROS has detrimental consequences on cell viability through extensive damage of DNA, proteins and organelles [29]. Antioxidant N-acetyl-L-cysteine (NAC) was considered as an important molecule of antioxidant defense for cellular resistance to exogenous stress [30, 31]. Thus, antioxidant N-acetyl-L-cysteine (NAC) was employed as positive control to verify whether creatine could maintain hypoxic TNBC cell survival. Addition of NAC or creatine to hypoxic TNBC cells could markedly decrease the level of proapoptotic proteins, including Bax and cleaved Caspase-3, and increased the level of anti-apoptosis protein Bcl-2 and cell viability associated protein Ki-67 (Fig. 7a), as further proved by correspondingly increased hypoxic tumor cell survival (Figure S6A), suggesting that the cytoprotective effects of creatine was partly due to its antioxidant capabilities. Cells with mitochondrial respiration deficiency due to exposure to hypoxia were reported to exhibit significant activation of AKT, conferring survival advantages on cells in hypoxia [32]. And it has been also reported that ROS-dependent repression of AKT-ERK signaling is involved in ROS-mediated cell death in various cancer cells [33]. To further decipher the potential molecular mechanism, we examined the activation of AKT and ERK upon administration of creatine or NAC to hypoxic TNBC cells. As expected, the levels of activated (phosphorylated) AKT and ERK were increased by addition of creatine or NAC in hypoxic TNBC cells (Fig. 7b). To find out whether creatine affects cell viability via AKT/ERK signaling, we treated cells with AZD5363 (an AKT inhibitor). Administration of AZD5363 to creatine-treated hypoxic breast cancer cells mitigated the effect of creatine on activation of AKT-ERK signaling (Fig. 7c), and correspondingly led to decrease of Bcl-2 protein and increase of pro-apoptotic proteins (e.g. Bax and activated (cleaved) Caspase-3) (Fig. 7d), which was echoed by corresponding alteration of hypoxic TNBC cell viability (Figure S6B). Collectively, these data demonstrate that creatine plays a preventative role to intracellular oxidative stress, thus to promote TNBC cell survival via activating AKT-ERK1/2 signaling under hypoxic conditions.

### Creatine reduces oxidative stress and facilitates TNBC tumor growth in vivo

**Fig. 8:**
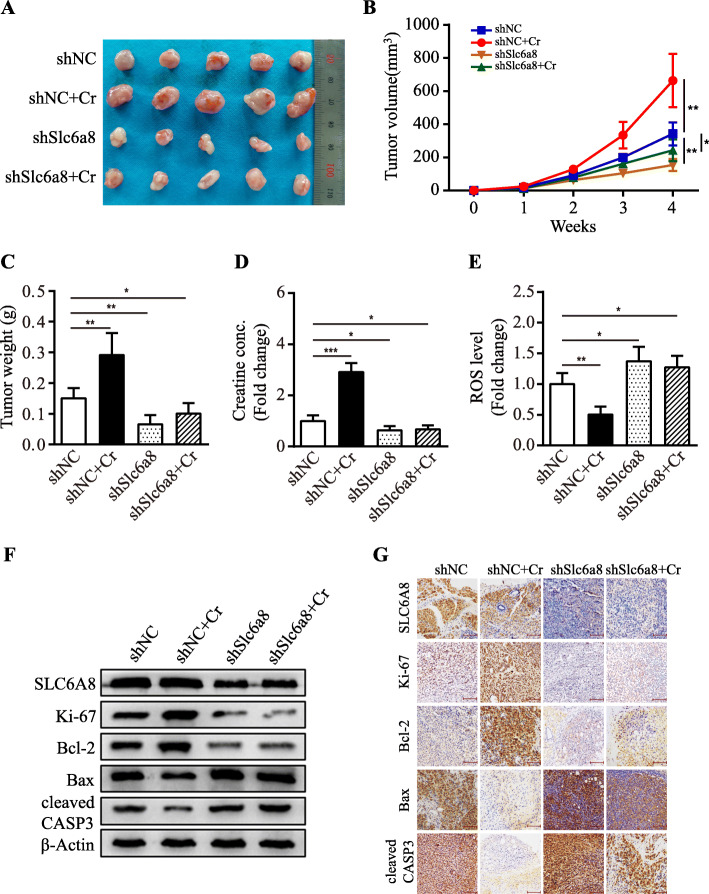
Enhanced creatine promotes tumorigenesis and attenuates oxidative stress in vivo. **a** Photos of the xenograft tumors. **b** Orthotopic tumor growth curve measured on a weekly basis. **c** The average tumor weight of each group was shown. **d** Average creatine concentrations of xenograft tumors. **e** Relative ROS levels were detected in tumor tissues by DCFH-DA staining. **f, g** SLC6A8, Ki-67, Bcl-2, Bax and cleaved Caspase-3 (cleaved CASP3) protein levels in tumor tissues were determined by western blot and IHC analysis (Scale bars, 100 μm). Data were presented as mean ± SD (^*^P < 0.05, ^**^P < 0.01, ^***^P < 0.001)

To expand our findings, SLC6A8-functional and loss-of-functional MDA-MB-231 cells were orthotopically inoculated into female nude mice to evaluate the tumor-promoting properties of creatine in vivo. In line with our in vitro results, the mice injected with SLC6A8-functional tumor cells (MDA-MB-231/shNC) had bigger tumor burden than the ones injected with SLC6A8-silenced tumor cells (MDA-MB-231/sh*Slc6a8*), and exogenous addition of creatine could notably promote tumor growth in SLC6A8-functional tumors rather than in SLC6A8-functional loss tumors (Fig. 8a-c). Consistently, larger amount of intratumoral creatine and lower ROS levels were detected in the orthotopic xenograft tumors derived form SLC6A8-functional MDA-MB-231 cells than those from SLC6A8-functional loss MDA-MB-231 cells (Fig. 8d-e). Checked by IHC staining and western blot, creatine levels showed a significantly positive correlation with the expression of SLC6A8, Bcl-2 and Ki-67, and was inversely correlated to Bax and cleaved Caspase-3 abundances (Fig. 8f-g). In summary, hypoxia-induced upregulation of SLC6A8 promotes intratumoral creatine accumulation and preserves cellular redox homeostasis, which augments TNBC cell survival and tumor growth via activating AKT/ERK signaling cascade.

## Discussion

A growing body of evidence has shown a crucial role of hypoxia in modulating cancer cell metabolic adaptation during tumor malignant progression [34]. However, it is not well understood regarding the precise molecular mechanisms. Here, our current work pinpoints a novel mechanism by which hypoxia improves cellular redox status to facilitate TNBC cell survival. We uncover that creatine is enriched through upregulation of SLC6A8 by p65/NF-κB in response to hypoxia and promotes cell survival via attenuation of mitochondrial complex I activity, reduction of OCR and ultimately prevention of an excess of ROS formation. Moreover, we highlight the clinical relevance of SLC6A8 in TNBC progression for the first time.

As an inherent characteristic of solid tumors, hypoxia causes a shortage of terminal electron acceptor (oxygen) during passage of electrons through the ETC and subsequently induces overproduction of ROS, which is cell-destructing when exceeding a certain threshold [35]. Abnormally elevated ROS can oxidize lipids, carbohydrates and proteins, leading to cellular dysfunction, senescence, pyroptosis, apoptosis or ferroptosis [36]. Compared to normal cells, tumor cells exhibit aberrant antioxidant systems, including increased synthesis of antioxidants, overexpressed antioxidant genes and augmented NADPH generation to keep ROS levels at a dynamic range that evades cellular death [37]. It has been documented that NDUFA4L2 is upregulated to decrease mitochondrial activity to prevent ROS accumulation, which inhibits apoptosis in HCCs under hypoxia [38]. Besides, activity of glucose-6-phosphate dehydrogenase (G6PD), the rate-limiting enzyme of the pentose phosphate pathway (PPP), was reported to be activated in response to hypoxia in human lung cancers [39]. Elevated PPP flux is implicated in several human cancers and was demonstrated to provide NADH and GSH for maintaining redox balance and supporting cancer cell survival under oxidative stress [40]. Here, we reveal a novel molecular mechanism of tumor cells maintaining appropriate mitochondrial ROS levels under hypoxia, that is, hypoxia activated p65/NF-κB signaling induces expression of SLC6A8 and creatine accumulation to upregulate antioxidant capacity in TNBC cells, which provides a new mechanistic insight into the control of oxidative stress in hypoxic tumor cells. And the current work also underscores that creatine presents potential metabolic vulnerabilities for targeting tumors that express high level of SLC6A8. Moreover, the enhanced SLC6A8 is common in several other solid tumors, implying the cytoprotective effects of creatine in hypoxic solid tumors as well.

Our study provides the first evidence that creatine exerts antioxidant capacities in TNBC cells under hypoxia. Conventionally, creatine has been best known for its role as an energy buffer, which confers a selective survival advantage to several cancer cells, including colorectal cancer cells and leukemia cells. For instance, HCT116 colon cancer cells upregulated creatine metabolism to stabilize cellular ATP levels for survival in response to hypoxia and HIF1 blockade [41]. Lately, creatine has been proved to exert direct or indirect antioxidant effects in various cells. It has been reported that creatine is able to directly to scavenge reactive oxygen species in living cells [42]. Indirectly, creatine has been shown to be able to protect mitochondrial DNA from oxidative attacks in HUVEC cells [43], protect against oxidative RNA damage in T leukemia cells [44], and induce antioxidant enzymes peroxiredoxin-4 and thioredoxin in C2C12 cells [45]. However, the mechanism underlying the antioxidant effects of creatine in breast cancer has yet to be clarified. Here, we unveil that creatine acts as an antioxidant through blunting mitochondrial complex I activity, which diminishes excessive ROS production in TNBC cells under hypoxia. Oxygen is reduced by electrons into water at the mitochondrial complexes of ETC, so the electron flow along the ETC is disturbed under hypoxia condition, thus leading to ROS accumulation [46]. Of note, mitochondrial complex I is a dominate source of mitochondrial ROS [47]. Accumulating studies have revealed that genetic abnormalities including mutations or alteration of expressions of genes in mitochondria are implicated in respiratory chain complex I deficiency and reduction of complex I activity [48]. Under hypoxia condition, there are multiple changes in the composition or activities of electron transport chain (ETC) complexes, which reduces electron flow through ETC and prevents excessive ROS formation. For example, NDUFA4L2, a component of ETC complex I, is upregulated to decrease mitochondrial activity to prevent excessive ROS formation under hypoxia [49]. In the current study, we also prove that complex I activity reduction by creatine plays an essential role in diminishing excessive ROS production in breast cancer cells.

Usually, cancer cells of solid tumors undergo oxidative stress as compared to normal cells [50]. To avoid senescence, apoptosis or ferroptosis that would be triggered by excessive ROS formation, cancer cells booster their antioxidant capacities to tolerate high ROS levels [51, 52]. Key redox-sensitive transcription factors such as Nuclear Factor, Erythroid 2-Like 2 (NRF2) are activated in response to oxidative stress and involved in regulating the expression of genes related with intracellular redox status [53]. For example, oxidative PPP is often turned on due to NRF2-regulated Transketolase (TKT) overexpression to counteract oxidative stress in cancer cells [54]. Dimethylaminomicheliolide (DMAMCL) was shown to induce the generation of ROS, resulting in inhibition of the AKT pathway and induction of apoptosis in HCCs [55]. Here, our work unveils that reduction of ROS by creatine can activate AKT-ERK1/2 signaling to upregulate Ki-67 and Bcl-2 expressions and downregulate Bax and cleaved Caspase-3 expressions, thus leading to enhanced cell survival under hypoxia.

## Conclusions

**Fig. 9:**
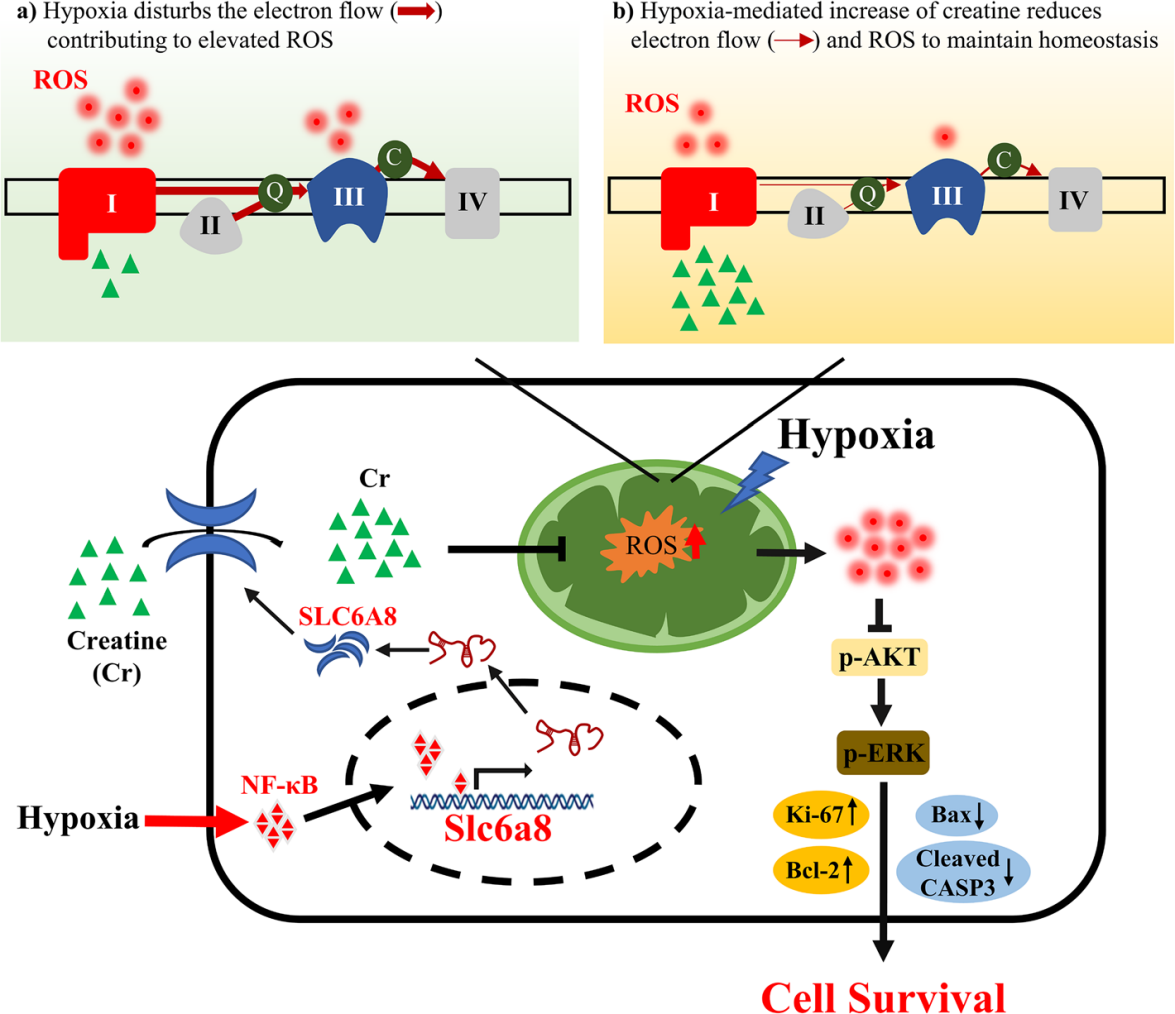
Schematic diagram of the protective role of creatine in hypoxic TNBC cells. The schematic diagram illustrating that SLC6A8 is transcriptionally upregulated by p65/NF-κB, thus mediating accumulation of intracellular creatine and maintaining cellular redox balance to promote TNBC cell survival via activating AKT-ERK1/2 signaling under hypoxia

To summarize, SLC6A8-mediated accumulation of creatine increases antioxidant capabilities of hypoxic TNBCs. The accumulation of creatine blunts mitochondrial complex I activity and decreases OCR to prevent excessive ROS production under hypoxia. The reduced ROS activates AKT-ERK signaling to upregulate pro-survival proteins including Ki-67 and Bcl-2, and downregulate pro-apoptosis proteins of Bax and cleaved Caspase-3, thus playing an essential role in promoting TNBC cell survival (Fig. 9). Our work addresses a new mechanistic insight into the regulation of TNBC metabolic reprogramming by hypoxia and might offer implications for improved TNBC therapy.

## Supplementary Information

**Additional file 1 Supplementary Figure 1.** Verification of the RNA-seq analysis. **a, b.** Expressions of 15 obviously upregulated RNAs (**a**) and 15 significantly upregulated RNAs encoding solute carrier transporters (**b**) were randomly selected and detected in MDA-MB-231 cells that were cultured under normoxia or hypoxia for 24 h by qRT-PCR. **Supplementary Figure 2.** Analysis of *Slc6a8* expression in various solid tumors. Box and whisker plots on *Slc6a8* mRNA expression levels in various tumor tissues, including bladder cancer (BC), colorectal cancer (CRC), esophageal squamous cell carcinoma (ESCC), clear cell renal cell carcinoma (CRCC), squamous cell lung carcinoma (SCLC) and melanoma, and their normal counterparts based on analysis of Oncomine database. mRNA levels were presented as log2 median-centered ratio. **Supplementary Figure 3.** The upregulation of SLC6A8 expression is not mediated by HIF1A/2A in hypoxic TNBC cells. **a, c.** HIF1A was stably knocked down by HIF1A-directed shRNA, and the RNA and protein levels of both HIF1A and SLC6A8 in shNC and shHIF1A hypoxic MDA-MB-231 and BT549 cells were detected by qRT-PCR and western blot, respectively. **b, d.** HIF2A was stably silenced by HIF2A-directed shRNA, and the RNA and protein levels of both HIF2A and SLC6A8 in shNC and shHIF2A hypoxic MDA-MB-231 and BT549 cells were determined by qRT-PCR and western blot, respectively. **e, f.** The Pearson correlation analysis of *Slc6a8* and *HIF1A* (**e**) or *HIF2A* (**f**) levels in TNBC based on TCGA database. Data were presented as mean ± SD (^**^P < 0.01). **Supplementary Figure 4.** SLC6A8 is upregulated by p65/NF-κB in hypoxic TNBC cells. **a**. A Venn diagram depicting the overlap between hypoxia-responsive transcriptional factors (TFs) and TFs of *Slc6a8* predicted by promo alggen database and JASPAR. **b-d**. Pearson correlation analysis of *Slc6a8* and *TP53* (**b**), *FOS* (**c**) or *ETV4* (**d**) levels based on TCGA database. **e**. qRT-PCR was performed to check TP53, p65/NF-κB, FOS and ETV4 RNA expression in MDA-MB-231 and BT549 cells cultured under normoxia or hypoxia condition for 24 h. **Supplementary Figure 5.** Creatine reduces mitochondrial activity, oxygen consumption and ROS production in hypoxic TNBC cells. **a.** Representative fluorescent images of JC-1 staining in shNC and shSlc6a8 TNBC cells exposed to normoxia (Norx) and hypoxia (Hypx) (Scale bars, 50 μm). **b.** Representative fluorescent images of DCFH-DA staining in shNC and shSlc6a8 TNBC cells exposed to normoxia (Norx) and hypoxia (Hypx) (Scale bars, 50 μm). **Supplementary Figure 6.** The enhanced creatine promotes hypoxic TNBC survival via attenuation of ROS-depended repression of AKT-ERK signaling. **a.** Cell viability was measured in hypoxic TNBC cells supplemented with creatine or NAC. **b.** Cell treated with creatine alone or combined with AKT inhibitor AZD5363 under hypoxia, and cell viability was detected. Data were presented as mean ± SD (^*^P < 0.05, ^**^P < 0.01).

**Additional file 2: Supplementary Table 1.** List of hairpin sequences used in the study. **Supplementary Table 2.** List of primer sequences utilized in the study.

## Data Availability

All data that can support the conclusions of this article are included in the article.

## Abbreviations

TNBC: Triple-negative breast cancer
SLC6A8: Solute carrier family 6 member 8, CT1
HER2: Human epidermal growth factor receptor 2
qRT-PCR: Quantitative real-time PCR
TNM stage: Tumor-node-metastasis stage
FBS: Fetal bovine serum
ROS: Reactive oxygen species
TF: Transcription factor
p65/NF-κB: Nuclear factor kappa B p65 subunit
AKT: Protein kinase B
ERK1/2: Extracellular regulated protein kinases
DCFH-DA: 2,7-Dichlorodihydrofluorescein diacetate
HIF1A: Hypoxia inducible factor 1 subunit alpha
HIF2A: Endothelial PAS domain protein 1
MMP: Mitochondrial membrane potential
NAC: N-acetyl-L-cysteine
OCR: Oxygen consumption rate
OXPHOS: Oxidative phosphorylation
PPP: Pentose phosphate pathway
Bax: BCL2-associated X
Bcl-2: B-cell lymphoma-2
Caspase-3: Apoptosis-related cysteine peptidase
CI: NADH–ubiquinone oxidoreductase
CIII: Ubiquinol–cytochrome c reductase
ETC: Electron transport chain
IHC: Immunohistochemistry
TCGA: The Cancer Genome Atlas
