# Supplementary material for: SLC6A8-mediated intracellular creatine accumulation enhances hypoxic breast cancer cell survival via ameliorating oxidative stress
Source: J Exp Clin Cancer Res. 2021 May 14;40:168. doi: 10.1186/s13046-021-01933-7 (PMC8120850; doi:10.1186/s13046-021-01933-7)
Supplement: Supplementary file 1 — Additional file 1 Supplementary Figure 1. Verification of the RNA-seq analysis. a, b. Expressions of 15 obviously upregulated RNAs (a) and 15 significantly upregulated RNAs encoding solute carrier transporters (b) were randomly selected and detected in MDA-MB-231 cells that were cultured under normoxia or hypoxia for 24 h by qRT-PCR. Supplementary Figure 2. Analysis of Slc6a8 expression in various solid tumors. Box and whisker plots on Slc6a8 mRNA expression levels in various tumor tissues, including bladder cancer (BC), colorectal cancer (CRC), esophageal squamous cell carcinoma (ESCC), clear cell renal cell carcinoma (CRCC), squamous cell lung carcinoma (SCLC) and melanoma, and their normal counterparts based on analysis of Oncomine database. mRNA levels were presented as log2 median-centered ratio. Supplementary Figure 3. The upregulation of SLC6A8 expression is not mediated by HIF1A/2A in hypoxic TNBC cells. a, c. HIF1A was stably knocked down by HIF1A-directed shRNA, and the RNA and protein levels of both HIF1A and SLC6A8 in shNC and shHIF1A hypoxic MDA-MB-231 and BT549 cells were detected by qRT-PCR and western blot, respectively. b, d. HIF2A was stably silenced by HIF2A-directed shRNA, and the RNA and protein levels of both HIF2A and SLC6A8 in shNC and shHIF2A hypoxic MDA-MB-231 and BT549 cells were determined by qRT-PCR and western blot, respectively. e, f. The Pearson correlation analysis of Slc6a8 and HIF1A (e) or HIF2A (f) levels in TNBC based on TCGA database. Data were presented as mean ± SD (**P < 0.01). Supplementary Figure 4. SLC6A8 is upregulated by p65/NF-κB in hypoxic TNBC cells. a. A Venn diagram depicting the overlap between hypoxia-responsive transcriptional factors (TFs) and TFs of Slc6a8 predicted by promo alggen database and JASPAR. b-d. Pearson correlation analysis of Slc6a8 and TP53 (b), FOS (c) or ETV4 (d) levels based on TCGA database. e. qRT-PCR was performed to check TP53, p65/NF-κB, FOS and ETV4 RNA expression in MDA-MB-231 and BT549 [file 13046_2021_1933_MOESM1_ESM.pptx]

## Slide 1
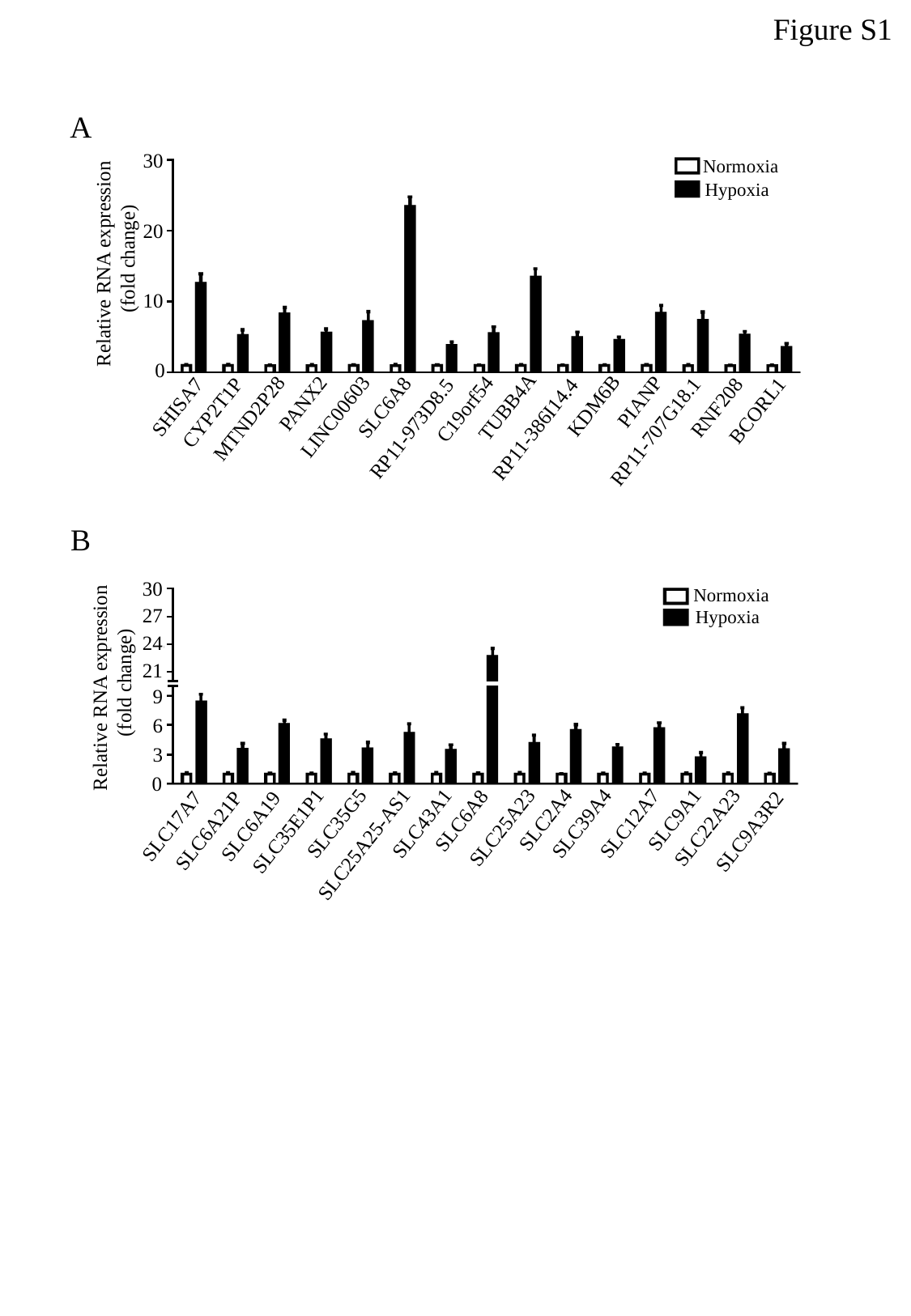

Figure S1
A
30
Normoxia
Hypoxia
20
Relative RNA expression
 (fold change)
10
0
PIANP
PANX2
KDM6B
SHISA7
TUBB4A
RNF208
SLC6A8
C19orf54
BCORL1
CYP2T1P
LINC00603
MTND2P28
RP11-973D8.5
RP11-386I14.4
RP11-707G18.1
B
30
Normoxia
27
Hypoxia
24
21
Relative RNA expression
 (fold change)
9
6
3
0
SLC2A4
SLC9A1
SLC6A8
SLC35G5
SLC43A1
SLC39A4
SLC12A7
SLC17A7
SLC6A19
SLC25A23
SLC22A23
SLC6A21P
SLC35E1P1
SLC9A3R2
SLC25A25-AS1

## Slide 2
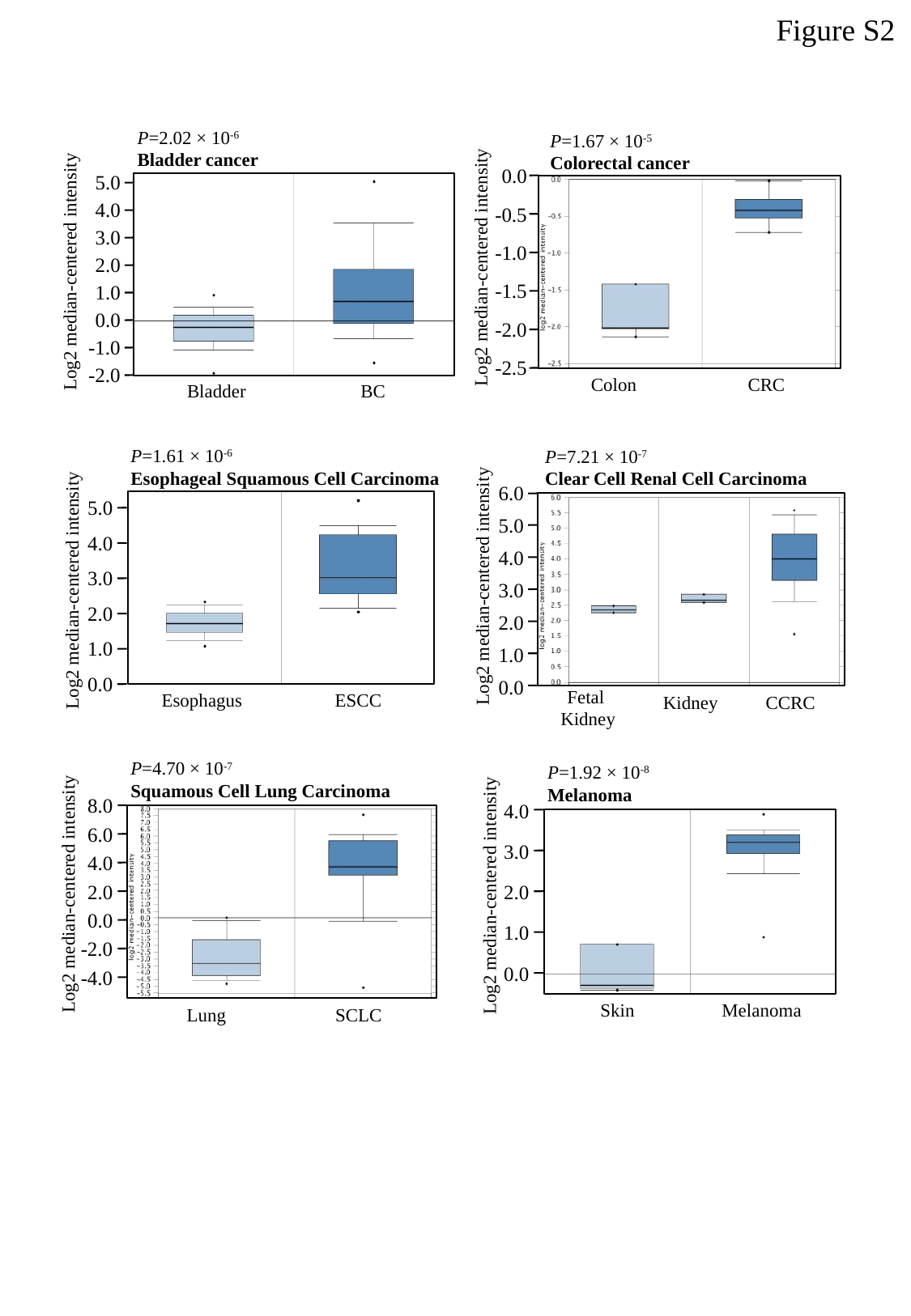

Figure S2
P=2.02 × 10^-6^
Bladder cancer
5.0
4.0
3.0
2.0
1.0
0.0
-1.0
-2.0
Log2 median-centered intensity
Bladder
BC
P=1.67 × 10^-5^
Colorectal cancer
0.0
-0.5
-1.0
Log2 median-centered intensity
-1.5
-2.0
-2.5
Colon
CRC
P=1.61 × 10^-6^
Esophageal Squamous Cell Carcinoma
5.0
4.0
3.0
2.0
1.0
0.0
Log2 median-centered intensity
Esophagus
ESCC
P=7.21 × 10^-7^
Clear Cell Renal Cell Carcinoma
6.0
5.0
4.0
3.0
2.0
1.0
0.0
Log2 median-centered intensity
Fetal
Kidney
Kidney
CCRC
P=4.70 × 10^-7^
Squamous Cell Lung Carcinoma
8.0
6.0
4.0
2.0
0.0
-2.0
-4.0
Log2 median-centered intensity
Lung
SCLC
P=1.92 × 10^-8^
Melanoma
4.0
3.0
2.0
1.0
0.0
Log2 median-centered intensity
Skin
Melanoma

## Slide 3
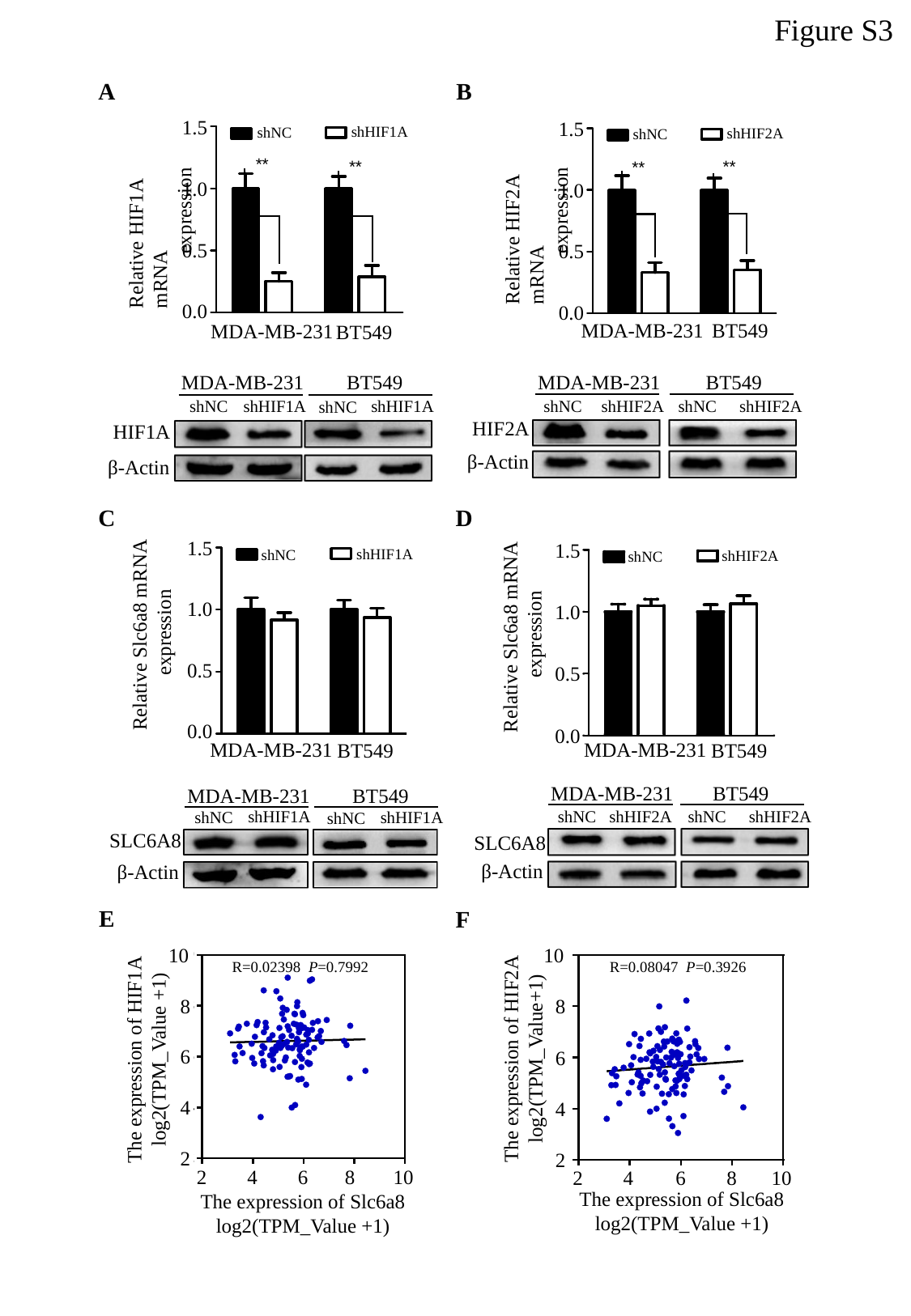

Figure S3
A
B
Relative HIF1A mRNA
 expression
1.5
1.0
0.5
0.0
shHIF1A
shNC
**
**
MDA-MB-231
BT549
Relative HIF2A mRNA
 expression
1.5
1.0
0.5
0.0
shHIF2A
shNC
**
**
MDA-MB-231
BT549
MDA-MB-231
BT549
shNC
shHIF2A
shNC
shHIF2A
HIF2A
β-Actin
MDA-MB-231
BT549
shHIF1A
shNC
shHIF1A
shNC
HIF1A
β-Actin
C
D
1.5
Relative Slc6a8 mRNA
 expression
shHIF1A
shNC
1.0
0.5
0.0
MDA-MB-231
BT549
1.5
Relative Slc6a8 mRNA
 expression
shHIF2A
shNC
1.0
0.5
0.0
MDA-MB-231
BT549
MDA-MB-231
BT549
shNC
shHIF2A
shNC
shHIF2A
SLC6A8
β-Actin
MDA-MB-231
BT549
shHIF1A
shNC
shHIF1A
shNC
SLC6A8
β-Actin
E
F
10
R=0.02398 P=0.7992
8
The expression of HIF1A
log2(TPM_Value +1)
6
4
2
2
4
6
8
10
The expression of Slc6a8
log2(TPM_Value +1)
10
R=0.08047 P=0.3926
8
The expression of HIF2A
log2(TPM_Value+1)
6
4
2
2
4
6
8
10
The expression of Slc6a8
log2(TPM_Value +1)

## Slide 4
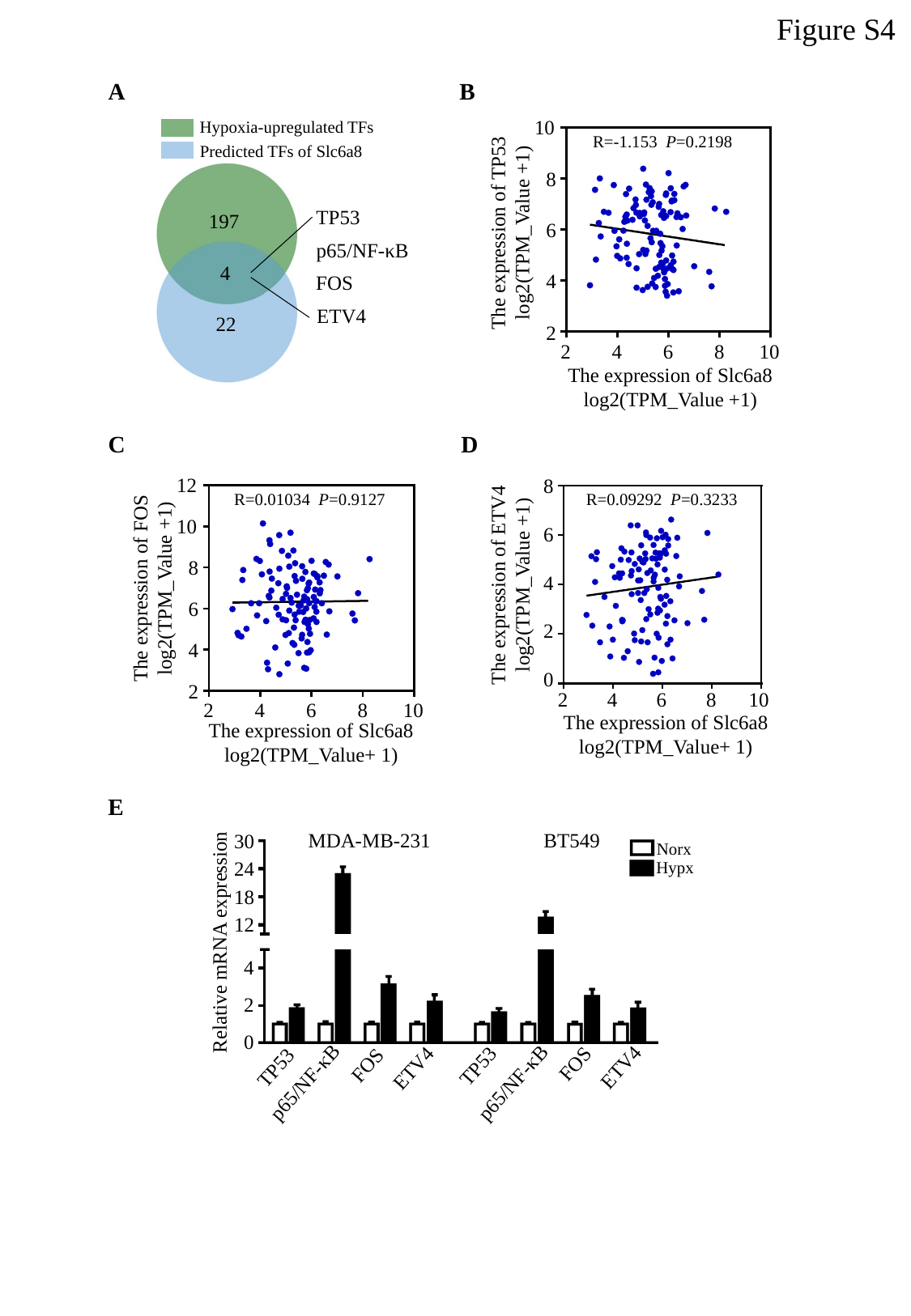

Figure S4
A
B
10
R=-1.153 P=0.2198
8
The expression of TP53
log2(TPM_Value +1)
6
4
2
8
2
4
6
10
The expression of Slc6a8
log2(TPM_Value +1)
Hypoxia-upregulated TFs
Predicted TFs of Slc6a8
197
4
22
TP53
p65/NF-κB
FOS
ETV4
C
D
12
R=0.01034 P=0.9127
10
8
The expression of FOS
log2(TPM_Value +1)
6
4
2
2
4
6
8
10
The expression of Slc6a8
log2(TPM_Value+ 1)
8
R=0.09292 P=0.3233
6
The expression of ETV4
log2(TPM_Value +1)
4
2
0
2
4
6
8
10
The expression of Slc6a8
log2(TPM_Value+ 1)
E
MDA-MB-231
BT549
30
Norx
24
Hypx
18
12
Relative mRNA expression
4
2
0
FOS
FOS
TP53
ETV4
TP53
ETV4
p65/NF-kB
p65/NF-kB

## Slide 5
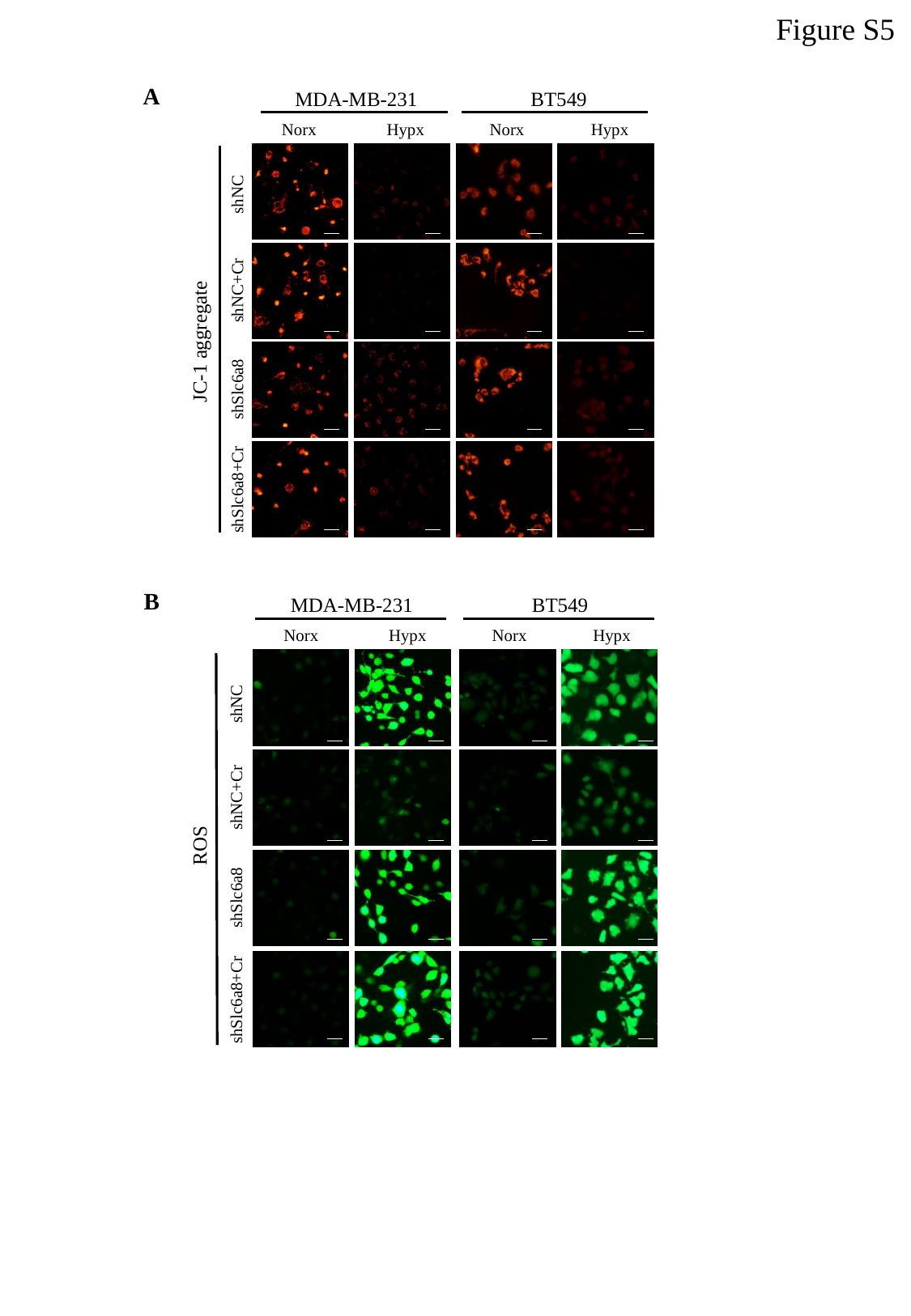

Figure S5
A
MDA-MB-231
BT549
Norx
Hypx
Norx
Hypx
shNC
shNC+Cr
JC-1 aggregate
shSlc6a8
shSlc6a8+Cr
B
MDA-MB-231
BT549
Norx
Hypx
Norx
Hypx
shNC
shNC+Cr
ROS
shSlc6a8
shSlc6a8+Cr

## Slide 6
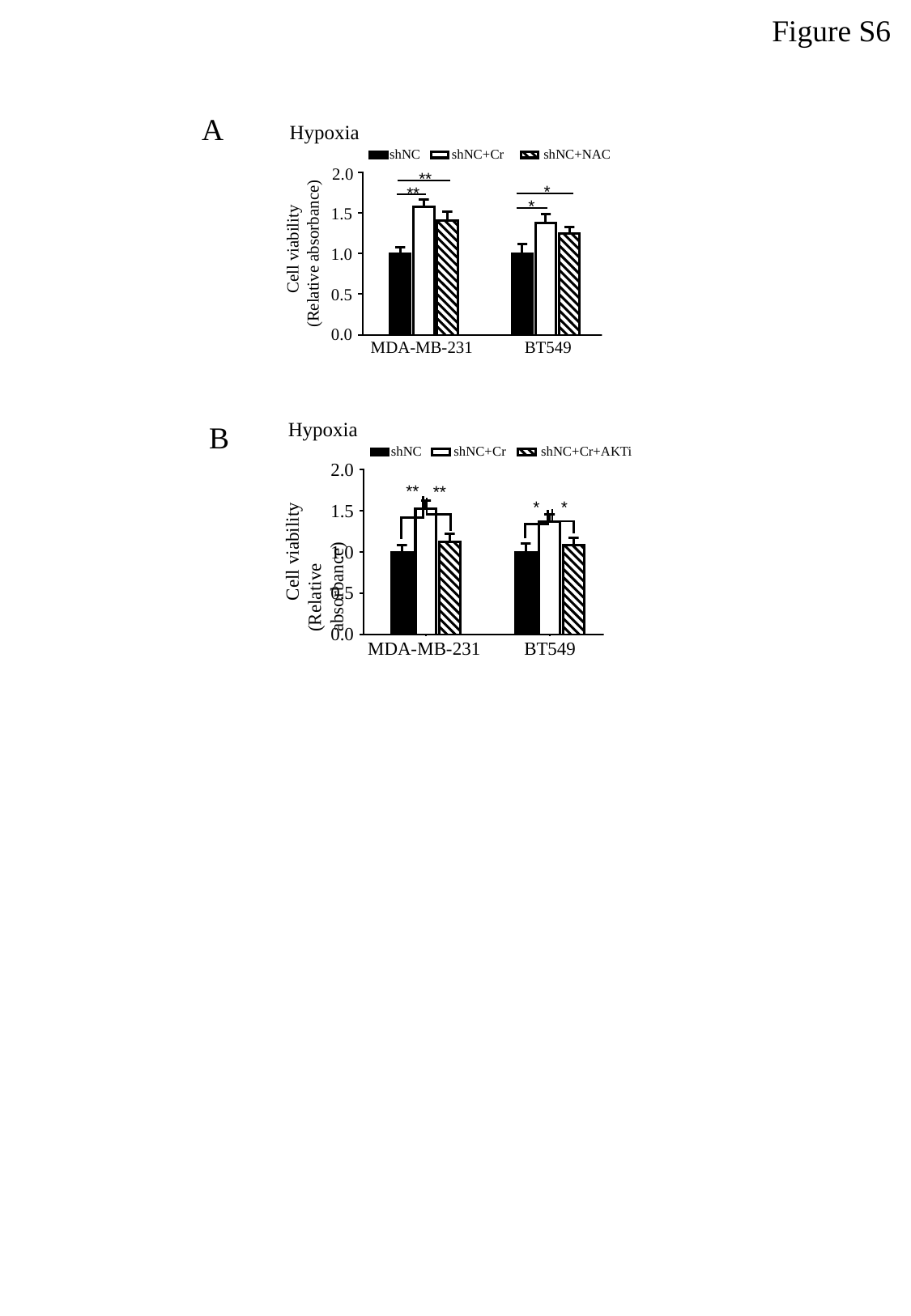

Figure S6
A
Hypoxia
shNC+Cr
shNC+NAC
shNC
2.0
**
*
**
*
1.5
Cell viability
(Relative absorbance)
1.0
0.5
0.0
MDA-MB-231
BT549
Hypoxia
shNC
shNC+Cr
shNC+Cr+AKTi
2.0
**
**
*
*
1.5
Cell viability
(Relative absorbance)
1.0
0.5
0.0
MDA-MB-231
BT549
B
