# Supplementary material for: SLC6A8-mediated intracellular creatine accumulation enhances hypoxic breast cancer cell survival via ameliorating oxidative stress
Source: J Exp Clin Cancer Res. 2021 May 14;40:168. doi: 10.1186/s13046-021-01933-7 (PMC8120850; doi:10.1186/s13046-021-01933-7)
Supplement: Supplementary file 2 — Additional file 2: Supplementary Table 1. List of hairpin sequences used in the study. Supplementary Table 2. List of primer sequences utilized in the study. [file 13046_2021_1933_MOESM2_ESM.pptx]

## Slide 1
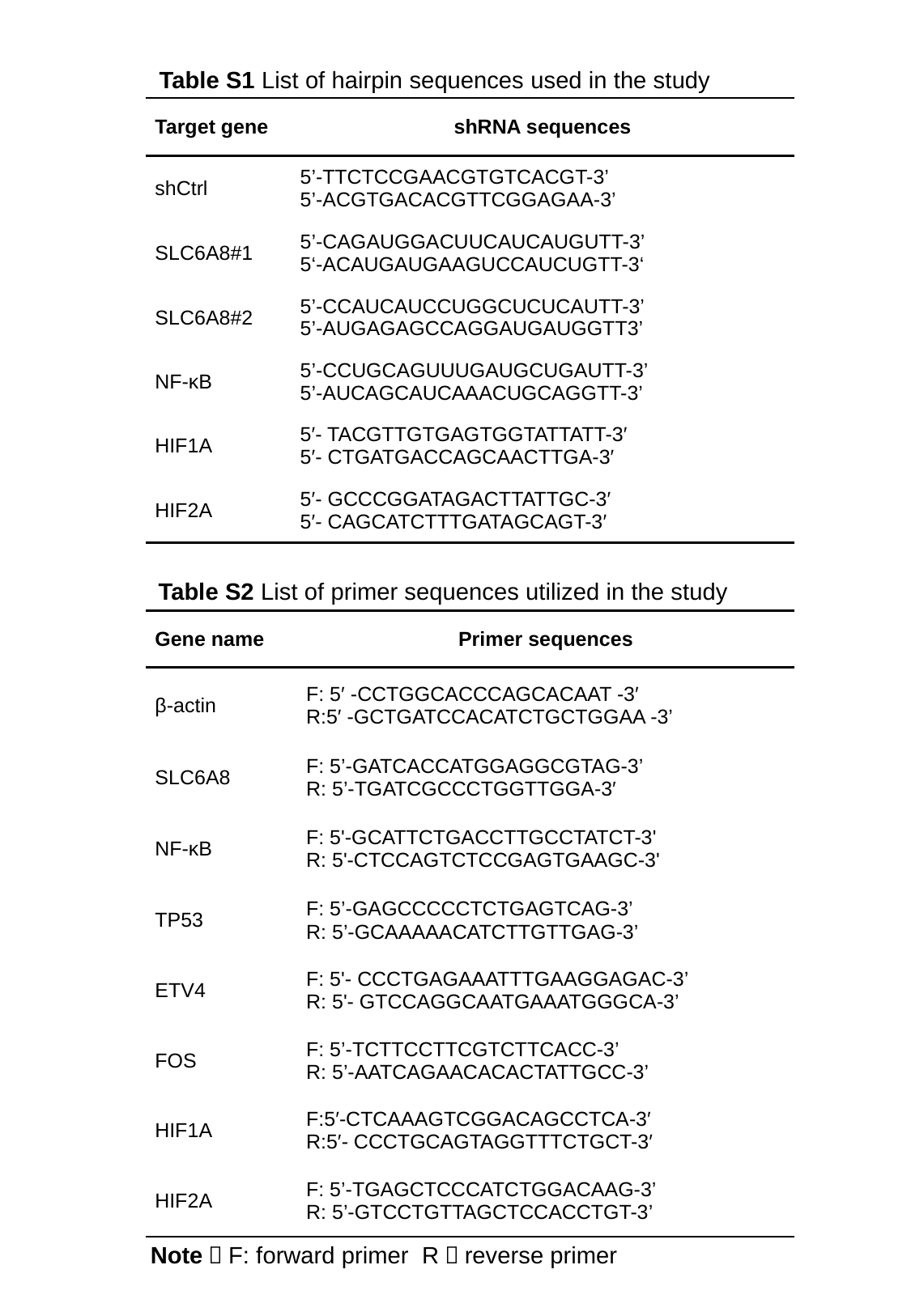

Table S1 List of hairpin sequences used in the study
| Target gene | shRNA sequences |
| --- | --- |
| shCtrl | 5’-TTCTCCGAACGTGTCACGT-3’ 5’-ACGTGACACGTTCGGAGAA-3’ |
| SLC6A8#1 | 5’-CAGAUGGACUUCAUCAUGUTT-3’ 5‘-ACAUGAUGAAGUCCAUCUGTT-3‘ |
| SLC6A8#2 | 5’-CCAUCAUCCUGGCUCUCAUTT-3’ 5’-AUGAGAGCCAGGAUGAUGGTT3’ |
| NF-κB | 5’-CCUGCAGUUUGAUGCUGAUTT-3’ 5’-AUCAGCAUCAAACUGCAGGTT-3’ |
| HIF1A | 5′- TACGTTGTGAGTGGTATTATT-3′ 5′- CTGATGACCAGCAACTTGA-3′ |
| HIF2A | 5′- GCCCGGATAGACTTATTGC-3′ 5′- CAGCATCTTTGATAGCAGT-3′ |
Table S2 List of primer sequences utilized in the study
| Gene name | Primer sequences |
| --- | --- |
| β-actin | F: 5′ -CCTGGCACCCAGCACAAT -3′ R:5′ -GCTGATCCACATCTGCTGGAA -3’ |
| SLC6A8 | F: 5’-GATCACCATGGAGGCGTAG-3’ R: 5’-TGATCGCCCTGGTTGGA-3′ |
| NF-κB | F: 5'-GCATTCTGACCTTGCCTATCT-3' R: 5'-CTCCAGTCTCCGAGTGAAGC-3' |
| TP53 | F: 5’-GAGCCCCCTCTGAGTCAG-3’ R: 5’-GCAAAAACATCTTGTTGAG-3’ |
| ETV4 | F: 5'- CCCTGAGAAATTTGAAGGAGAC-3’ R: 5'- GTCCAGGCAATGAAATGGGCA-3’ |
| FOS | F: 5’-TCTTCCTTCGTCTTCACC-3’ R: 5’-AATCAGAACACACTATTGCC-3’ |
| HIF1A | F:5′-CTCAAAGTCGGACAGCCTCA-3′ R:5′- CCCTGCAGTAGGTTTCTGCT-3′ |
| HIF2A | F: 5’-TGAGCTCCCATCTGGACAAG-3’ R: 5’-GTCCTGTTAGCTCCACCTGT-3’ |
Note：F: forward primer R：reverse primer
